# Gifsy-1 Prophage IsrK with Dual Function as Small and Messenger RNA Modulates Vital Bacterial Machineries

**DOI:** 10.1371/journal.pgen.1005975

**Published:** 2016-04-08

**Authors:** Tal Hershko-Shalev, Ahuva Odenheimer-Bergman, Maya Elgrably-Weiss, Tamar Ben-Zvi, Sutharsan Govindarajan, Hemda Seri, Kai Papenfort, Jörg Vogel, Shoshy Altuvia

**Affiliations:** 1 Department of Microbiology and Molecular Genetics, IMRIC, The Hebrew University-Hadassah Medical School, Jerusalem, Israel; 2 Institute for Molecular Infection Biology, University of Würzburg, Würzburg, Germany; University of Geneva Medical School, SWITZERLAND

## Abstract

While an increasing number of conserved small regulatory RNAs (sRNAs) are known to function in general bacterial physiology, the roles and modes of action of sRNAs from horizontally acquired genomic regions remain little understood. The IsrK sRNA of Gifsy-1 prophage of *Salmonella* belongs to the latter class. This regulatory RNA exists in two isoforms. The first forms, when a portion of transcripts originating from *isrK* promoter reads-through the IsrK transcription-terminator producing a translationally inactive mRNA target. Acting in *trans*, the second isoform, short IsrK RNA, binds the inactive transcript rendering it translationally active. By switching on translation of the first isoform, short IsrK indirectly activates the production of AntQ, an antiterminator protein located upstream of *isrK*. Expression of *antQ* globally interferes with transcription termination resulting in bacterial growth arrest and ultimately cell death. *Escherichia coli* and *Salmonella* cells expressing AntQ display condensed chromatin morphology and localization of UvrD to the nucleoid. The toxic phenotype of AntQ can be rescued by co-expression of the transcription termination factor, Rho, or RNase H, which protects genomic DNA from breaks by resolving R-loops. We propose that AntQ causes conflicts between transcription and replication machineries and thus promotes DNA damage. The *isrK* locus represents a unique example of an island-encoded sRNA that exerts a highly complex regulatory mechanism to tune the expression of a toxic protein.

## Author Summary

As the function of conserved core-genome-encoded small RNAs (sRNA) reflects the basic lifestyle of bacteria, the function of non-conserved island-encoded sRNAs remains enigmatic. The island-encoded sRNA IsrK belongs to Gifsy-1 prophage of *Salmonella*. Here, we report a complex mechanism in which the IsrK RNA functions as both sRNA and mRNA to control the production of the toxic AntQ protein. The *isrK* promoter directs the synthesis of two distinct RNA species: a full-length translationally inactive target mRNA and the correctly terminated, shorter IsrK sRNA. IsrK sRNA binds the full-length inactive mRNA producing an antiterminator protein, AntQ, which interferes with transcription termination. Expression of *antQ* results in bacterial growth arrest and ultimately cell death. Fluorescence microscopy of *E*. *coli* and *Salmonella* expressing *antQ* revealed condensed chromatin morphology as observed upon exposure to DNA-damaging agents. We propose that expression of the phage antiterminator protein results in conflicts between transcription and replication machineries and thus facilitates DNA damage. In summary, the RNA regulator IsrK presents a new regulatory principle in which a horizontally acquired sRNA controls genome integrity.

## Introduction

The first systematic searches for bacterial sRNA were based on bio-computational identification of conserved genes in intergenic regions [1]. The subsequent characterization of these conserved sRNAs identified them as important players in many adaptive and physiological responses. These conserved core-genome encoded regulatory sRNAs comprise many antisense RNAs of which a subset are *cis*-encoded whereas the majority acts on *trans*-encoded target mRNAs by limited base complementarity [2]. Most *trans*-acting base-pairing sRNAs of enteric bacteria require the RNA chaperone protein Hfq for both intracellular stability and for efficient annealing to target mRNAs [3]. However, the chromosomes of these bacteria are mosaics, composed of conserved collinear regions interspersed with unique genetic islands that were acquired horizontally via once-mobile genetic elements. Therefore, the early searches based on sequence conservation generally disregarded unique horizontally acquired sRNAs. However, subsequent global cDNA cloning, comparative genomic based expression screens, Hfq-bound and global transcriptomic screens detected short RNA species in non-conserved horizontally acquired regions as well as highly abundant short RNA species from the UTRs of protein-coding genes [4–8]. The function of these non-conserved sRNAs remains enigmatic yet promising, as they may inform new regulatory principles.

Members of the genus *Salmonella* carry numerous genomic islands that were acquired by horizontal transfer of phages, plasmids and transposons. These islands carry fitness and virulence genes that are integral to *Salmonella* pathogenicity, enabling the bacteria to adapt to different niches, invade intestinal cells and multiply within cells of the immune response [9]. In a previous study, we screened the horizontally acquired genomic islands of *Salmonella typhimurium* for non-conserved sRNA genes. Our analysis led to identification of 19 unique island-encoded sRNAs including the Gifsy-1 prophage encoded IsrK RNA [6]. The chromosome of *Salmonella* is lysogenic for a number of phages including Gifsy-1 and Gifsy-2, both of which carry genes implicated in *Salmonella* virulence [9, 10]. Bacteriophage Gifsy-2 carries the *sodCI* gene encoding a periplasmic superoxide (Cu,Zn)-dismutase, with a proposed role in *Salmonella* defense against killing by macrophages as well as a number of genes encoding type III secreted effectors [11–13]. Gifsy-1 carries multiple virulent factors such as *gipA*, which is involved in bacterial colonization of small intestine [14, 15]. To coordinate expression between core and island genes, bacteria often recruit sRNAs [16, 17]. For example, InvR from the major SPI-1 island represses the production of the core-encoded major outer membrane protein OmpD [18]. IsrE, the paralogue of RyhB, represents another example of a cross talk between genes of core and islands [6]. The island-encoded sRNA IsrE is regulated by Fur, a core-encoded repressor in response to iron-deplete conditions and contributes to control of the core-encoded iron regulon [6, 19]. Here we show that IsrK of Gifsy-1 prophage controls the expression of its genetic locus leading to growth arrest of *Salmonella* by acting as small and messenger RNA, in an Hfq-independent manner. The growth inhibition is caused by an increase in expression of a Q-like antiterminator protein (here denoted AntQ) that is encoded on the same locus.

AntQ belongs to Q proteins’ family of lambdoid phages. Bacteriophage λ Q protein is an operon specific transcription anti-termination factor required for expression of the phage late genes. Q protein joins the elongation complex at early stages of transcription and enables RNA polymerase to read-through the terminator located upstream of the phage late genes [20–23]. To join the elongation complex, Q interacts with a specific DNA sequence element as well as with RNA polymerase that is paused during early elongation. The binding of Q alters the functional properties of the transcription elongation complex interfering with termination signals [24, 25]. We find that although Q proteins are known to bind specific sites within phages, the function of the Gifsy-1 Q-like protein AntQ is not limited to *Salmonella* or phage DNA. By contrast AntQ promotes transcription elongation of core genome transcripts resulting in growth arrest and ultimately cell death.

## Results

### The *isrK* genetic locus

**Fig 1:**
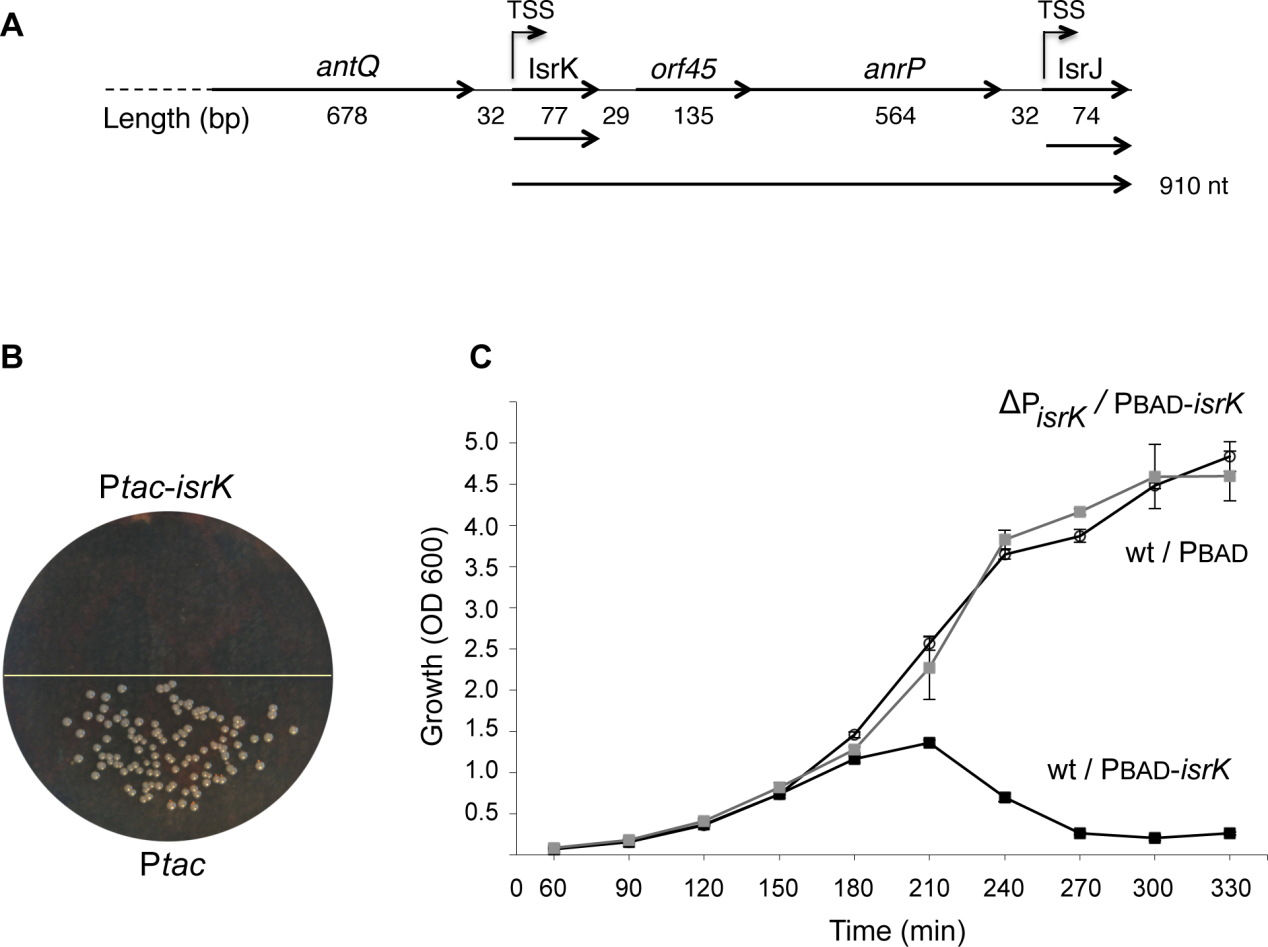
High levels of IsrK RNA inhibit growth of *Salmonella* wild type cells. (A) Schematic representation of the *isrK* locus. Transcription start sites are indicated (TSS). Horizontal arrows indicate stable RNA species. The two short species represent the previously characterized IsrK and IsrJ sRNAs, terminated at their *rho*-independent transcription termination sites. A subset of *cis* IsrK transcripts read-through the transcription termination signal and a polycistronic mRNA of about 910 nt is formed. (B) Transformation assay. *Salmonella* wild type was transformed with P*tac* plasmid and P*tac-isrK* expressing IsrK constitutively. (C) Growth curves of wild type *Salmonella* cells and cells deleted for the *isrK* promoter in the chromosome (ΔP*isrK* in grey) carrying a control plasmid (PBAD) or an *isrK* expressing plasmid (PBAD-*isrK*). To induce expression of *isrK*, arabinose (0.2%) was added at dilution. OD600 values were measured at indicated times.

The gene encoding IsrK sRNA is located within Gifsy-1 prophage. Upstream of IsrK is SL2579 encoding a Q-like anti-terminator protein (here denoted AntQ). The transcription start-site of *antQ* was mapped 1,600 bases upstream of *antQ* [8]. Downstream of *isrK* we noticed a putative ORF of 45 amino acids, followed by SL2578 encoding a predicted anti-repressor-like protein (denoted AnrP) and the previously identified sRNA encoding gene, *isrJ* (Fig 1A) [6]. To examine whether transcription of the downstream gene *anrP* is linked to IsrK, we constructed *isrK*-*orf45*-*anrP*-*lacZ* transcriptional fusions with and without the *isrK* promoter. The assays showed that IsrK promoter directs transcription of the downstream gene *anrP*. Interestingly, the corresponding translation fusion demonstrated that although the operon is transcribed, there is no translation of *anrP* mRNA (Table 1A).

**Table 1:** *anrP* expression regulation in response to IsrK sRNA expressed in *trans* (β-galactosidase activity).

A. Basal transcription of anrP*				
Genetic elements	Transcription	Translation		
P*isrK*-*isrK-orf45-anrP'-lacZ*	280±50	4±3		
*isrK-orf45-anrP'-lacZ*	3±0	4±0		
B. *anrP* expression regulation in response to IsrK sRNA expressed in *trans*				
P*isrK*-*isrK-orf45-anrP'-lacZ*	PBAD	PBAD-IsrK	Fold increase	
Transcription fusion*	230±20	1771±127	8	
Translation fusion**	3±1	417±66	139	
C. *anrP* translation activation by IsrK depends on *orf45* translation***				
Genetic elements	PBAD	PBAD-IsrK	Fold increase	
P*isrK*-*isrK-orf45-anrP'-lacZ*	4±1	456±53	114	
*orf45-*A107C (first AUG)^a^	3±0	61±10	20	
*orf45-*UG120-121AA (second AUG)^b^	16±2	624±14	39	
*orf45-*A107C G121A (both AUGs)^c^	3±0	68±15	23	
*orf45-*AU119-120UA (stop)^d^	11±2	92±9	8	
D. Mutations affecting structure A and/or interaction with IsrK****				
Genetic elements	PBAD	PBAD-IsrK	Fold	
P*isrK*-*isrK-orf45-anrP'-lacZ*	4±1	683±86	171	
*isrK-*G28A	93±27	1090±88	12	
*orf45-*C162U	484±36	1473±99	3	
*isrK-*G28A *orf45-*C162U	224±4	641±34	3	
*isrK-*G31A	218±2	1835±120	8	
*orf45-*C159U	926±81	1827±118	2	
*isrK-*G31A *orf45-*C159U	327±13	2656±155	8	
E. Mutations affecting the interaction with IsrK*****				
Genetic elements	PBAD	PBAD-IsrK	Fold	PBAD-IsrK*-*C18Ue	Fold
P*isrK*-*isrK-orf45-anrP'-lacZ*	3±0	365±21	121	245±15	81
*orf45-*G173A^e^	16±0	341±137	21	576±15	36
F. *cis* and *trans* effects of IsrK mutants****					
Genetic elements	PBAD	PBAD-IsrK	PBAD-IsrK*-*G28A	PBAD-IsrK*-*G31A	
P*isrK*-*isrK-orf45-anrP'-lacZ*	4±1	796±44	2±3	1±1	
*isrK-*G28A	93±27	1090±88	89±35	-	
*isrK-*G31A	218±2	1835±120	-	-	

aThe first AUG is now CUG

bThe second AUG is now AAA

cThe first and the second AUGs are now CUG and AUA, respectively

dThe second AUG is now UAG stop codon

eMutations G173A disrupts the intra-molecular structure and affect basepairing with wild type IsrK. G173A is complementary to IsrK-C18U.

*Average (miller units) of two independent assays

**Average (miller units) of 14 independent assays

***Average (miller units) of 4 (wild type) and four (mutants) independent assays.

****Average (miller units) of 4 (wild type) and two (mutants) independent assays.

*****Average (miller units) of two (wild type) and two (mutants) independent assays.

### High levels of IsrK RNA inhibit growth of *Salmonella*

We noticed that plasmid borne constitutive expression of *isrK* is toxic. *Salmonella* cells fail to yield any colonies when transformed with plasmids expressing *isrK*, constitutively (Fig 1B). To further investigate the toxic phenotype, the *isrK* gene was cloned under the inducible PBAD promoter and we followed bacterial growth in wild type and a strain deficient of the chromosomal *isrK* promoter. High levels of IsrK expressed in *trans* result in growth arrest of wild type *Salmonella* (Fig 1C). The growth inhibition is not observed when the chromosomal *isrK* promoter is deleted, suggesting that the genetic regulation leading to toxicity requires expression of the *isrK* locus in both *cis* and *trans*. In addition, we examined whether the IsrK-dependent toxicity involves lysis of the host by Gifsy-1 induction. To this end, we deleted Gifsy-1 genes SL2575 and SL2576 encoding proteins of phage lysis and phage lysozyme superfamily, respectively. Both mutant strains failed to yield any colonies when transformed with plasmids expressing *isrK* constitutively, indicating that the growth arrest phenotype does not involve Gifsy-1 induction and lysis of the host (S1 Fig).

### The toxic phenotype of IsrK is caused by AntQ, a Q-like anti-terminator protein

Deletion mapping at the *isrK* locus to identify the cause of toxicity demonstrated that strains deleted for sequences upstream (*antQ*) or downstream (*anrP*) of *isrK* showed no growth inhibition, forming normal size colonies when transformed with a plasmid expressing constitutive high levels of IsrK, indicating involvement of both genes (S2A Fig). To define whether any of the above-mentioned genetic elements is toxic when expressed alone, in the absence of IsrK, we transformed strains deleted of the locus including the lysis genes up to *antQ* with plasmids expressing *antQ* or *anrP* from P*tac* promoter under the control of the *lacI* repressor. Whereas cells transformed with plasmids expressing *anrP* formed regular colonies (S2B Fig), cells expressing *antQ* fail to grow, indicating that AntQ is sufficient for toxicity.

### IsrK mediated growth arrest correlates with *anrP* translation

The growth arrest data indicated that toxicity involves IsrK RNA present in *cis* and expressed in *trans*, hence, we monitored the effect of IsrK expressed in *trans* on transcription and translation at the *isrK* locus. A northern blot probed with an *isrK* specific primer, detected a long transcript of ~ 900 nucleotides when *isrK* was expressed in *trans* (S3A Fig). Probing the northern blot with an *isrJ* specific primer demonstrated that the long transcript encompasses *anrP*, suggesting that transcription starting at the *isrK* promoter reads-through the *isrK* Rho-independent transcription termination signal downstream into *orf45* and *anrP*. Analysis of shorter RNA species using *isrK* specific primer supports expression of the plasmid encoded *isrK* gene (S3B Fig, lanes 4–9), as well as chromosomally encoded short *isrK* form, indicating that transcription starting at the *isrK* promoter produces the IsrK sRNA as well as *isrK* operon mRNA (S3B Fig, lanes 1–3). In addition, a *lacZ*-transcription fusion starting at the *isrK* promoter (P*isrK*-*isrK-orf45-anrP'-lacZ*) showed that *isrK* expressed in *trans* increased the levels of the long transcript by ~8 fold. The *anrP* translation fusion also showed that *isrK* expressed in *trans* activates translation of *anrP* by 140 fold (Table 1B). Together these data demonstrate that RNA polymerase partially reads-through the *isrK* Rho-independent transcription termination signal downstream into *orf45*-*anrP* and that IsrK sRNA acting in *trans* causes a slight increase in the levels of the downstream polycistronic mRNA and activates *anrP* translation.

### Expression of *anrP* leads to an increase in *antQ* expression

**Fig 2:**
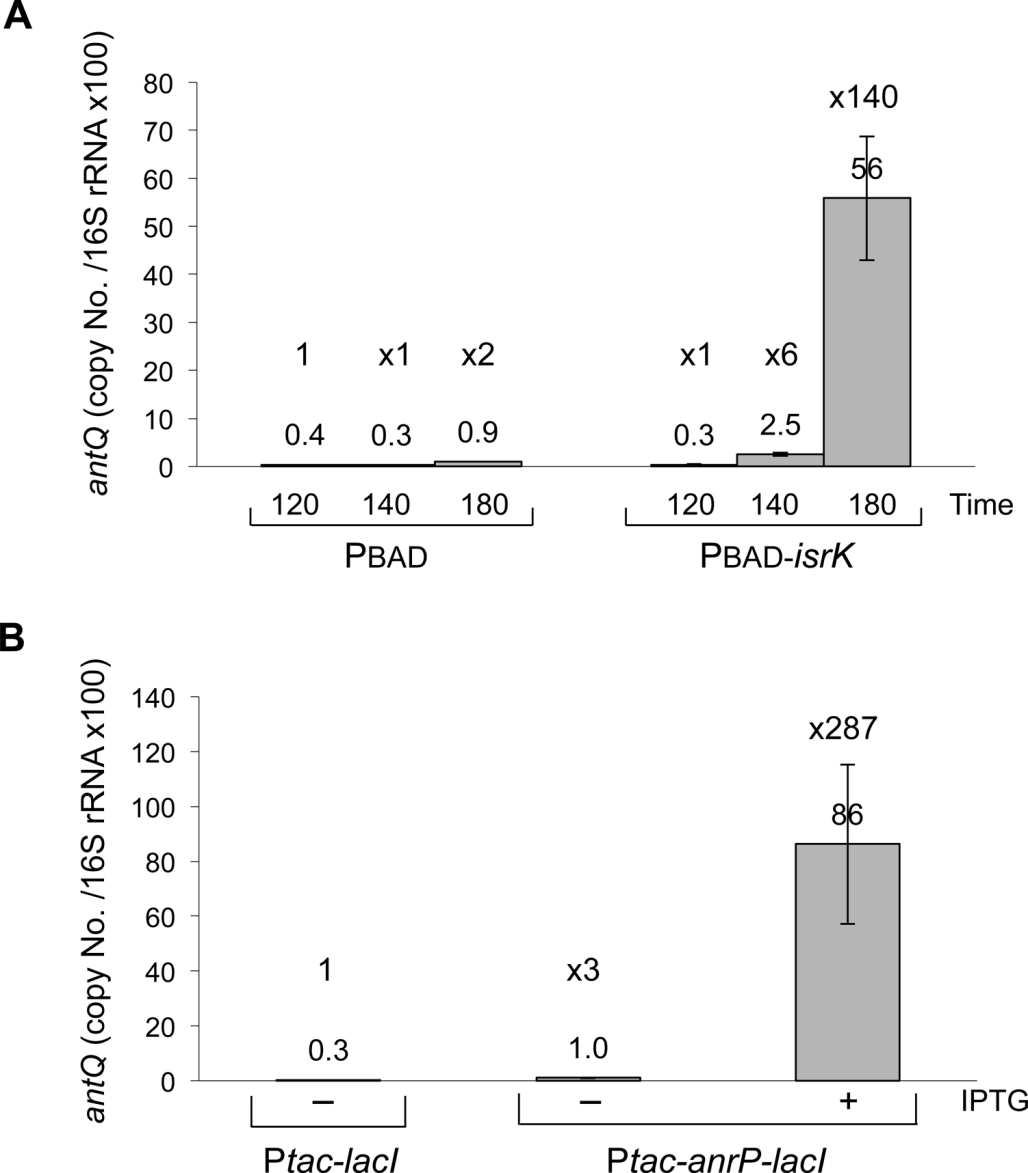
High-levels of IsrK or AnrP lead to an increase in expression of *antQ*. Real-Time PCR of *antQ* mRNA detected in the presence of high levels of IsrK (PBAD-*isrK*) (A) or AnrP (P*tac*-*anrP*-*lacI*) (B). *Salmonella* carrying control, *isrK*, and *anrP* expressing plasmids were exposed to arabinose and IPTG to activate PBAD and P*tac* promoters, respectively (see also Materials and Methods). Two samples per treatment and two reactions per sample were analyzed. We also measured the levels of the second gene of *antQ* operon, SL2581 (see S4 Fig).

Gifsy anti-repressor proteins bind to and inactivate the lysogenic repressor, thereby leading to transcription of phage operons [26]. To learn about the correlation between increased levels of IsrK sRNA, the anti-repressor protein AnrP and the anti-terminator AntQ, we monitored *antQ* mRNA levels upon expression of *isrK* and *anrP*, using quantitative Real-Time PCR. This analysis showed that in *trans* expression of *isrK* resulted in increased *antQ* mRNA levels. Similarly, in *trans* expression of *anrP* led to higher *antQ* transcript levels (Fig 2A and 2B). To learn about the activity of the anti-repressor protein AnrP, we measured RNA levels of SL2581, the second gene of *antQ* operon. Similarly to *antQ*, SL2581 levels increased upon expression of *isrK* or *anrP*, indicating that AnrP activates transcription of the *antQ* operon most likely through activated transcription activity (S4 Fig). Together, the results indicate that IsrK activates expression of *anrP*, which in turn leads to AntQ synthesis. Furthermore, we monitored Gifsy-1 prophage induction upon expression of *isrK* and *anrP* (S5 Fig). Phage plating on a susceptible strain demonstrates that Gifsy-1 phage induction by IsrK requires an intact *isrK* locus, whereas Gifsy-1 induction by AnrP is independent of *isrK* locus. These results further support the regulatory cascade we present for the *isrK* locus and the biological relevance of this locus to phage development. We also observed oxidative stress dependent general phage induction (Materials and Methods and S5C Fig), upon which the levels of IsrK sRNA increase during the first minutes of exposure to hydrogen peroxide while *antQ* and SL2581 mRNA levels increase gradually (S3D Fig and S6 Fig).

### IsrK induces expression of *anrP* via an upstream ORF

**Fig 3:**
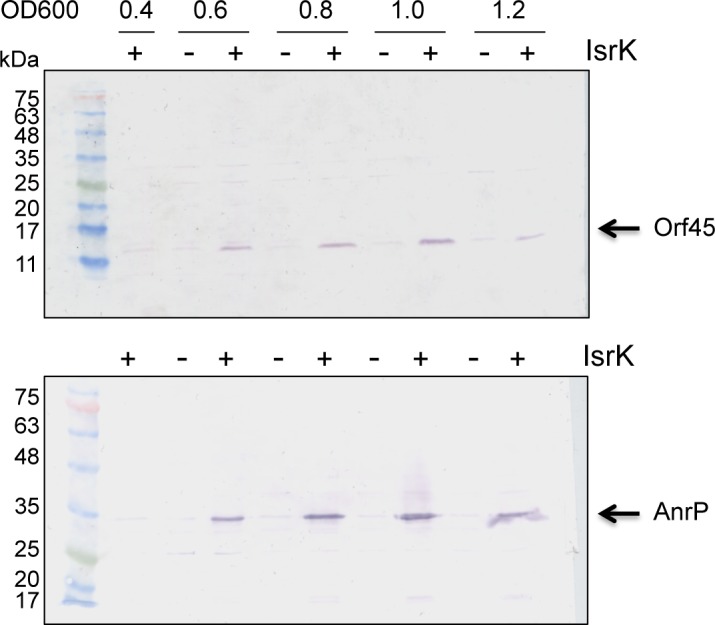
IsrK induces translation of *orf45* and *anrP*. Cells carrying the sequential peptide affinity (SPA) tag in the chromosome, adjacent to the carboxy-terminal amino acid of the *orf45* or *anrP* were transformed with control and *isrK* expressing plasmids. The cells were exposed to arabinose at the time of dilution and total protein was extracted at the indicated OD600. ORF 45 and AnrP were detected using SPA specific antibodies. The western shows that in *trans* expression of *isrK* increases translation of *orf45* and *anrP*.

To visualize the translation pattern of the downstream operon including *orf45* and *anrP*, we integrated the coding sequence of the sequential peptide affinity (SPA) tag [27] into *orf45* and *anrP* to generate C-terminal fusion proteins, in two separated strains. The western blot showed that IsrK expressed in *trans* increases translation of both *orf45* and *anrP* (Fig 3).

*orf45* carries two in frame initiation codons and a stop codon overlapping the initiation codon of *anrP* (AUGA). Nucleotide and amino acid conservation analysis demonstrated that the nucleotide sequence of *orf45* is conserved among the *enterobacteria* (S7 Fig), whereas the amino acid sequence of *orf45* varies (S8 Fig). The proximity of *orf45* to *anrP* prompted us to examine their potential translation coupling. By mutating the initiation codons we found that translation starting at the first initiation codon leads to translation of *anrP*. Moreover, insertion of a stop codon proximal to the translation initiation site reduced *anrP* translation activation by IsrK (Table 1C). Together, our data indicate that *isrK* expressed in *trans* and *orf45* translation are required to stimulate *anrP* translation.

### Alternative structures at the *isrK* locus

**Fig 4:**
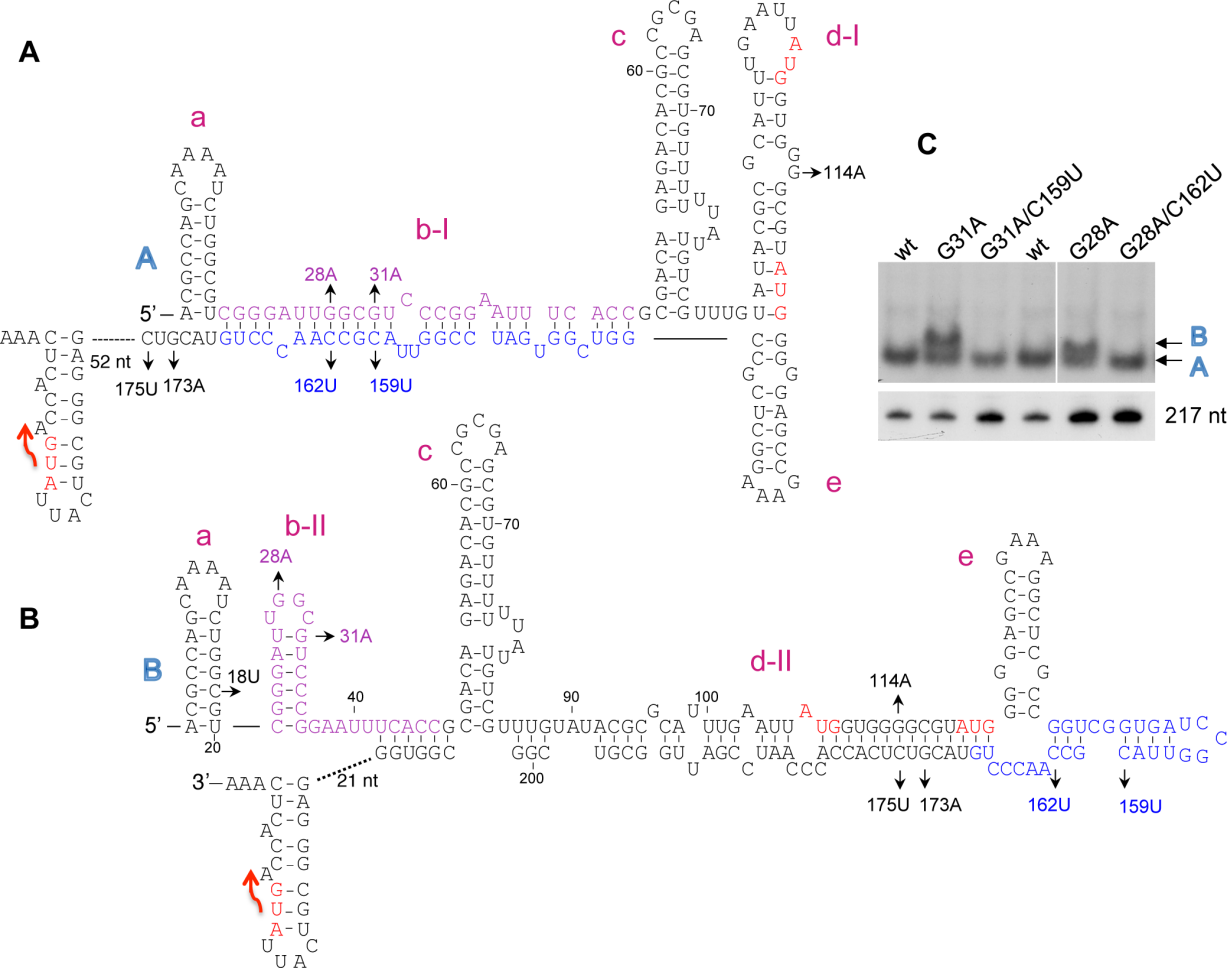
Alternative structures at the *isrK* locus. (A and B) The initiation codons of *orf45* and *anrP* are in red. The stop codon of *orf45* is marked by a red arrow. The helices (a to e) are marked in Magenta. In structure A, the middle part of IsrK (in purple) is engaged in helix b-I that is complementary to the 3’ end of *orf45* (in blue). In structure B, the purple sequence forms the middle hairpin of IsrK (b-II), whereas the *orf45* blue sequence is part of helix d-II. (C) Native gel analysis. (Top panel) *In vitro* synthesized *isrK-orf45* RNAs wild type and mutants as indicated were separated on non-denaturing polyacrylamide gels. The RNAs were detected using end-labeled primer complementary to sequences of *orf45*. Arrows indicate the two conformations observed. To verify the integrity of the RNAs, the samples were analyzed on denaturing 6% polyacrylamide and 7.8 M Urea gel (Bottom panel).

To investigate the mechanism of the translational regulation of *isrK*-*orf45*-*anrP* by IsrK, we induced random mutations at this locus using P*isrK-orf45-anrP-'lacZ* translation fusion plasmid and screened for high-level expression mutants in the absence of in *trans isrK*. Two high-level expression mutants were found to carry mutations within *isrK* (G28A and G31A). Structural prediction analysis using RNA fold program (http://rna.tbi.univie.ac.at/) show that the wild type transcript (1–180 nt) forms one conformation (A) having a ΔG of -85.66 kcal/mol, whereas the mutated RNA forms an alternative conformation (B) with a predicted ΔG value of -84.21 kcal/mol (Fig 4A and 4B). Functional studies of translation fusions carrying mutations G28A or G31A showed that G28A and G31A, which are predicted to form the alternative structure B, increase the basal level of AnrP translation (Table 1D), indicating that structure B is translationally active, whereas structure A is translationally silent. To visualize the two isoforms and to learn about their ratios in the wild type RNA, we examined the RNAs on nondenaturing polyacrylamide gels. The native gels demonstrate that wild type RNA is found almost exclusively in one structure, while G31A and G28A mutant RNAs display two conformers of which one resembles the wild type conformation and the other represents the alternative structure B (Fig 4C).

In the inactive structure formed by wild type RNA (A), the middle part of IsrK (in purple) base pairs with ~ 30 nt long sequence of *orf45* (in blue) forming helix b-I (Fig 4A and 4B). In this structure the ribosome-binding site of *orf45* forms hairpin d-I. In the alternative structure (B), the middle part of IsrK forms an alternative hairpin (b-II), whereas the RBS of *orf45* forms a new helix by pairing with its 3’-end (d-II). Mutations G28A and G31A are likely to destabilize structure A by disrupting the middle helix, but are predicted to have no effect on structure B. To examine base pairing, we modified helix b opposite to G28A and G31A to carry the corresponding complementary mutations C162U and C159U, respectively and when combined, would restore formation of the helix. RNA mutants carrying G28A/C162U or G31A/C159U exhibit one conformation, the same as wild type RNA (Fig 4A, 4B and 4C), indicating that G28A basepairing with C159U and G31A/C162U basepairing form structure A.

Functional studies of translation fusions carrying C162U and C159U showed that like mutations G28A or G31A, C162U and C159U mutants exhibited a high basal level of *anrP* translation (Table 1D). The basal levels decrease when these mutations are combined with the corresponding complementary mutations further indicating that structure A is translationally inactive, whereas structure B is translationally active. Because mutations C162U and C159U affect the stability of structure B in addition to A, they exhibit a higher basal level of translation than that observed for the opposite mutations (see below).

To affirm the differences between the two structures, we constructed mutations G114A and the corresponding complementary mutation C175U, both predicted to destabilize structure B with no effect on structure A. Given that wild type RNA is found almost exclusively in conformation A, these mutations only a mildly affected translation of *anrP* (S1 Table).

### IsrK sRNA binds *orf45* in *trans*

**Fig 5:**
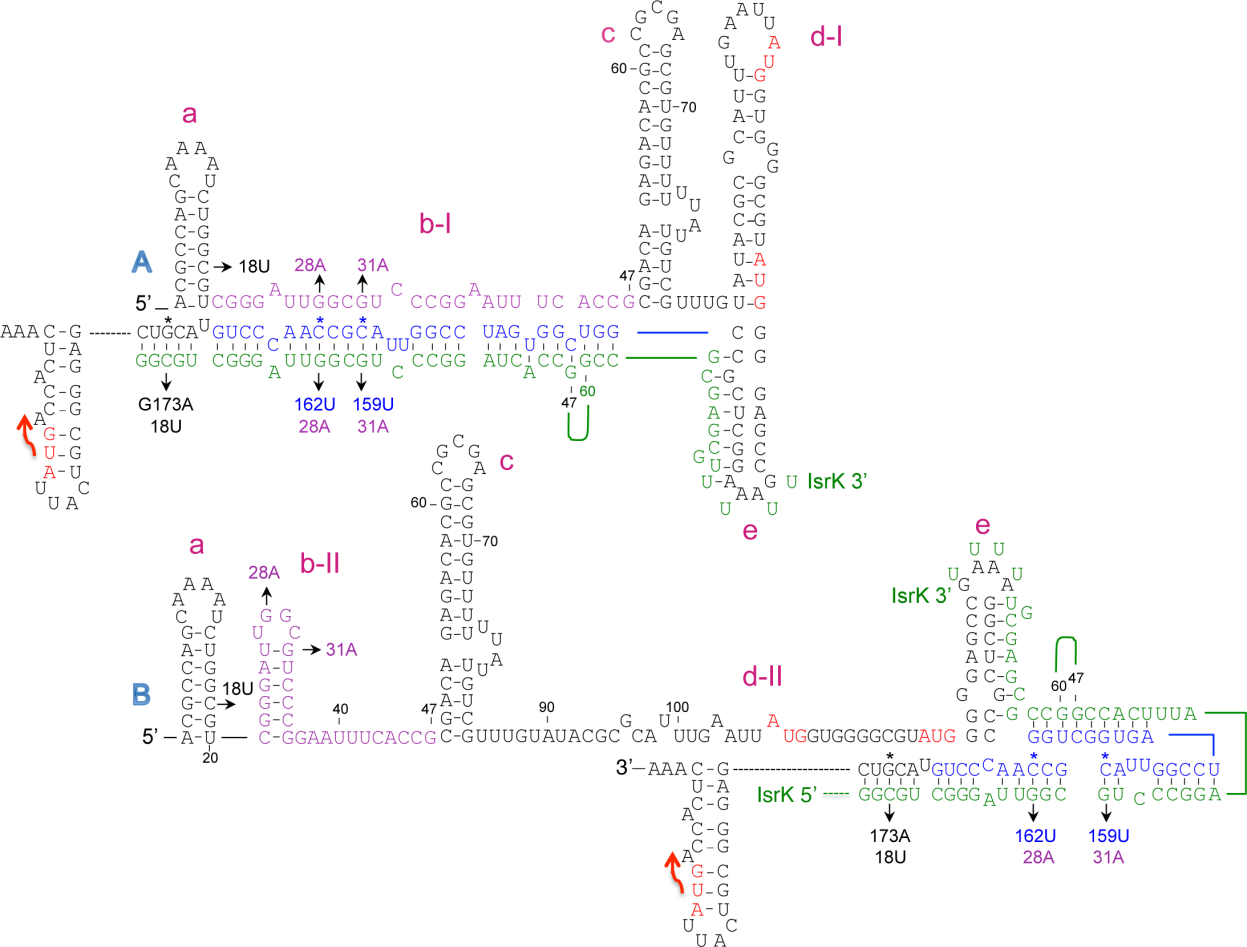
Base pairing of IsrK sRNA with the complementary sequence of *orf45*. In binding of structure A, IsrK (in green) is predicted to compete with its own sequence for the binding of the middle helix b-I. In structure B, binding of IsrK is predicted to destabilize helix d-II that sequesters the RBS of *orf45*. Nucleotides 47–60 of IsrK are not complementary to *orf45* (marked by an arc). Asterisks indicate mutations in the long transcript. The corresponding complementary mutations in IsrK are denoted below.

Fig 4A shows that in structure A, the middle part of the *cis*-encoded IsrK (in purple) binds a long sequence of *orf45* (in blue). Given the complementarity between *cis*-encoded IsrK and *orf45* and the influence of in *trans* expression of *isrK* on downstream translation, we explored the functional and structural consequences of IsrK binding to structures A and B, in *trans*. In binding to structure A, IsrK is predicted to compete with its own sequence for the binding of the middle helix (Fig 5). Binding of structure B by IsrK is predicted to destabilize the helix d-II that sequesters the RBS of *orf45* (Fig 5). Mutational analysis supported binding of in *trans* IsrK to the *cis*-encoded *isrK-orf45-anrP* target mRNA. Mutations C159U and C162U are predicted to affect base paring with IsrK by replacing CG pairs with UG pairs (Fig 5). Functional studies of translation fusions of C159U and C162U mutant RNAs demonstrate that wild type IsrK weakly affected translation of *anrP*, indicating that stable binding of IsrK in *trans* is important for *anrP* translation activation and that destabilization of the helix formed between in *trans* IsrK and *cis-*encoded *orf45* abrogates *anrP* translation control by IsrK (Table 1D). Similarly, mutation G173A is predicted to affect base paring with wild type IsrK by replacing a GC pair with an AC pair (Fig 5). Translation fusions studies of *orf45* carrying G173A mutation demonstrated that wild type IsrK RNA is less effective in activation of *anrP* translation than an IsrK mutant carrying the corresponding complementary mutation C18U (Table 1E). Likewise, mutation C175U replaces CG pair with UG pair and *anrP* translation activation by wild type IsrK is less productive. Moreover, because of imperfect basepairing, IsrK activation of G173A is lower than that of C175U mutant (S1 Table).

**Fig 6:**
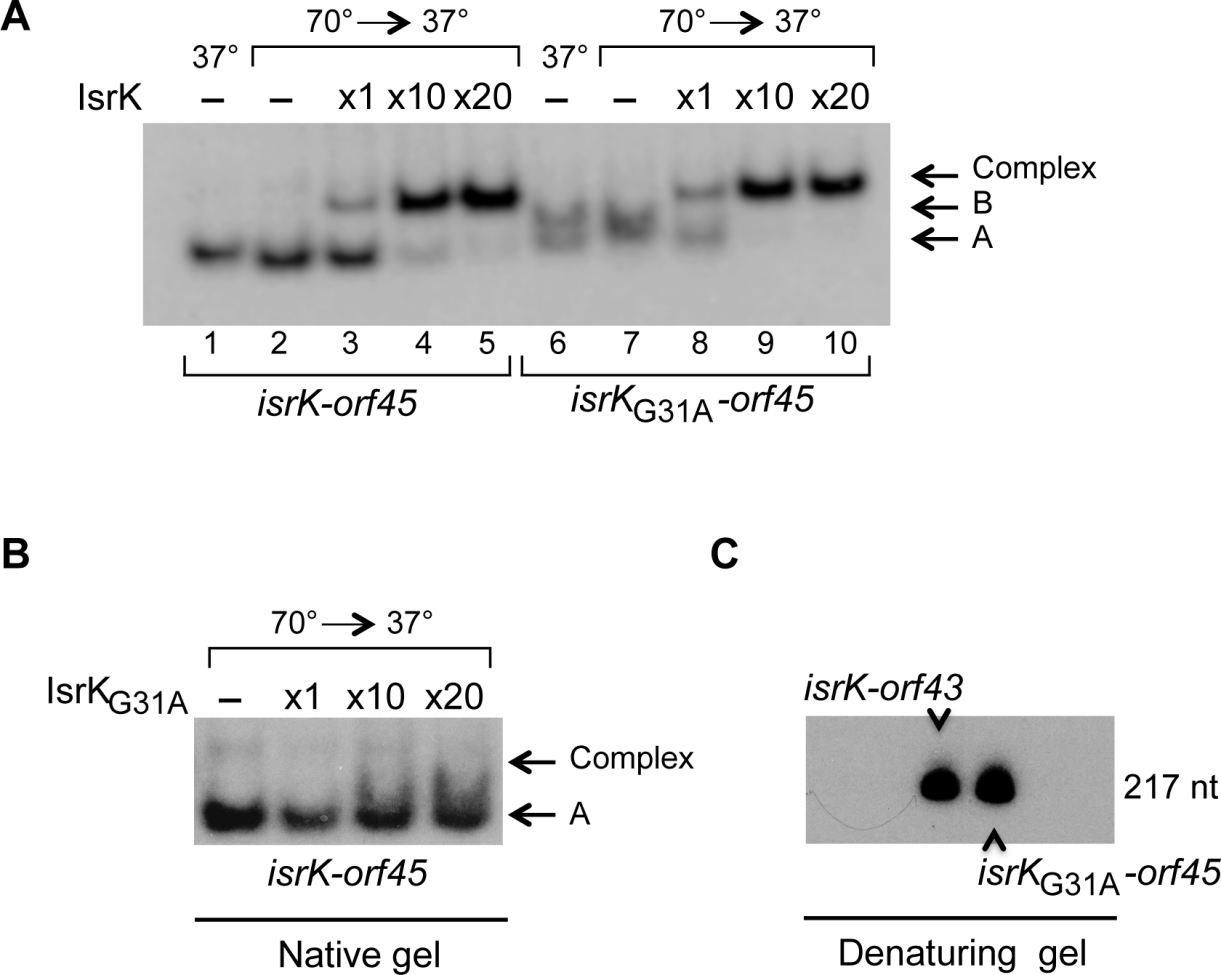
Binding of *isrK-orf45* by IsrK. (A) Target RNAs, *isrK*-*orf45* wild type and mutant were pre incubated at 37°C (lanes 1 and 6) or 70°C (lanes 2–5 and 7–10), chilled on ice and then incubated further without or with increasing amounts of IsrK at 37°C. Arrows indicate structures A and B and binding by IsrK (Complex). (B) Incubation of *isrK*-*orf45* wild type RNA with *isrK*G31A mutant results in no binding. (C) Analysis of the RNA samples on denaturing gels exhibits one form.

The effect of IsrK acting in *trans* is visible in native gels. Incubation of wild type RNA with IsrK at 37°C results in minimal binding of structure A to IsrK (S8 Fig). In accordance, binding of structure A of *isrK*-*orf45*G31A RNA by IsrK is indistinct as opposed to binding of the structure B of this RNA mutant (S8 Fig). Pre-incubation of the target RNAs at 70°C to denature the structures, prior to their incubation with IsrK, facilitates binding by IsrK. Under these conditions, IsrK binds structure A, characteristic of wild type RNA and both structure A and B that are characteristic of *isrK*-*orf45*G31A (Fig 6A). No binding can be detected when *isrK*-*orf45* wild type RNA is incubated with *isrK*G31A mutant further confirming that this mutant is inactive as supported by our cultivation experiments (Fig 6B, S10 Fig).

### IsrK facilitates 30S binding to *orf45* RBS

**Fig 7:**
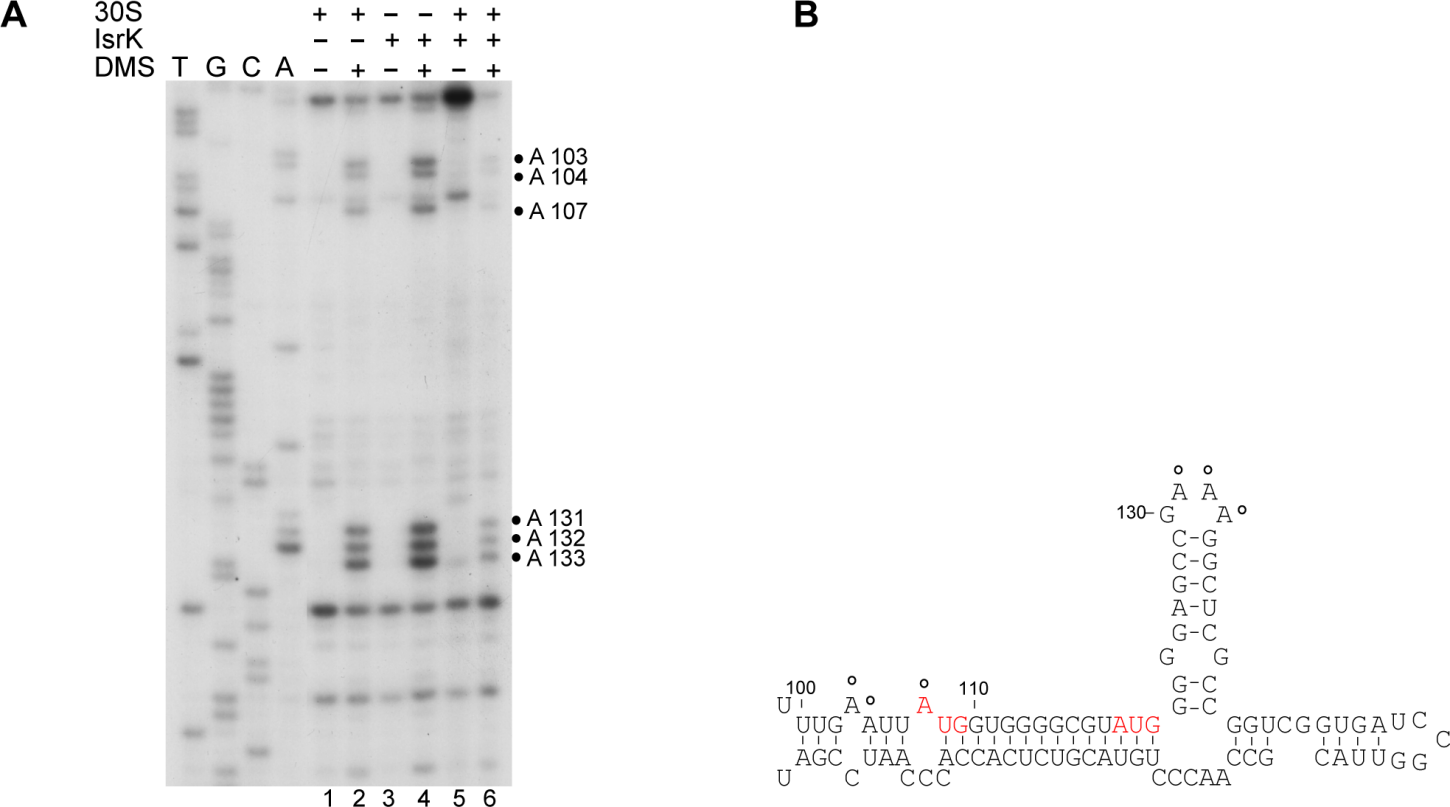
IsrK facilitates 30S binding to *orf45* RBS. (A) *In vitro* synthesized RNA templates were incubated with 30S ribosomes and/or IsrK RNA prior to the addition of DMS. Thereafter, the samples were treated with phenol as described in Materials and Methods. The modified sites were detected by primer extension. The numbers on the right indicate sequence positions relative to the transcription start site. (B) Open circles indicate modification sites protected by 30S binding. The initiation codons of *orf45* are indicated in red.

Since IsrK binds *orf45* adjacent to its RBS, detection of 30S binding in real time (toe printing) is inconceivable. However, 30S binding at the RBS would protect the neighboring upstream and downstream sequences. To probe the accessibility of the sequence surrounding the RBS, we used dimethyl sulfate (DMS), which methylates unpaired adenosine and cytidine residues at N1 and N3 positions, respectively. Samples were incubated with and without 30S and/or IsrK prior to the addition of DMS. The modified sites were detected by primer extension after phenol extraction. A few nucleotides that surround the RBS of *orf45* are susceptible to DMS modification in the presence of 30S ribosomes or IsrK (Fig 7 lanes 2,4). The same nucleotides are protected from DMS in presence of both IsrK and 30S (Fig 7 lane 6), indicating that wild type IsrK facilitates 30S binding to the RBS of *orf45*. 30S protection from DMS decreases much in the presence of *isrK*G31A mutant that is unable to bind *orf45* (S11 Fig).

### Dual *cis* and *trans* regulatory functions of *isrK* mutants

On the one hand, IsrK is part of the target *isrK*-*orf45*-*anrP* mRNA, and as such mutations in IsrK affect the structure this mRNA forms. For instance, G28A disrupts helix b in structure A, shifting the equilibrium towards the translationally active structure B. Therefore, mRNA carrying *isrK*G28A-*orf45*-*anrP* exhibits a high basal level of *anrP* translation. On the other hand, the short IsrK acts in *trans* to destabilize the *cis*-encoded translationally inactive target mRNA leading to *anrP* translation. Therefore, one mutation in *isrK* gene is predicted to yield two different phenotypes. We examined the effect of *isrK*G28A in *cis* and in *trans* and found that whereas, *cis*-encoded *isrK*G28A-*orf45*-*anrP* exhibits a high basal level of *anrP* translation; *isrK*G28A acting in *trans* is unable to activate *anrP* translation (Table 1F). In accordance, high levels of *isrK*G28A or *isrK*G31A expressed from the PBAD promoter have no effect on growth of wild type *Salmonella* (S10 Fig).

### AntQ toxicity is global, affecting the core genome

We have shown that toxicity involves expression control of *anrP* by IsrK that in turn induces transcription of the anti-terminator protein AntQ (Fig 2A and 2B). Considering the origin of *antQ*, *i*.*e*., Gifsy-1 prophage, we examined whether its toxicity is specific to *Salmonella* and/or phage genes. Accordingly, the influence of high levels of AntQ was investigated in two *E*. *coli* K-12 strains; wild type (MG1655) and MDS42 that is deleted of all genetic islands including prophages and insertion elements [28]. *antQ* expression repressed growth and decreased survival of both strains. At 40 minutes of induction, survival of wild type and MDS42 decreased by ~15 and ~30 fold, respectively, indicating that toxicity of the Q-like anti-terminator protein is not specific to *Salmonella* or phage DNA (S12 Fig). Moreover, the results suggest that AntQ protein has natural recognition sites within the core genome of these strains.

### AntQ mediated growth inhibition is rescued by Rho and SrmB

Bacteriophages Q antiterminator proteins interfere with transcription termination by binding specified sites at promoter regions and forming a persistent complex with RNA polymerase. This complex of RNA polymerase and Q protein can bypass terminators [24]. We examined changes in protein expression pattern of *Salmonella* upon exposure to AntQ using one-dimensional SDS-PAGE. Gel areas showing differences in the pattern of proteins because of AntQ were isolated and subjected to mass spectrometry (S13 Fig). Two proteins, whose expression increased, were selected for further analysis because of their score and annotation; the transcription termination factor Rho and the DEAD-box-containing ATP-dependent RNA helicase SrmB [29–32].

**Fig 8:**
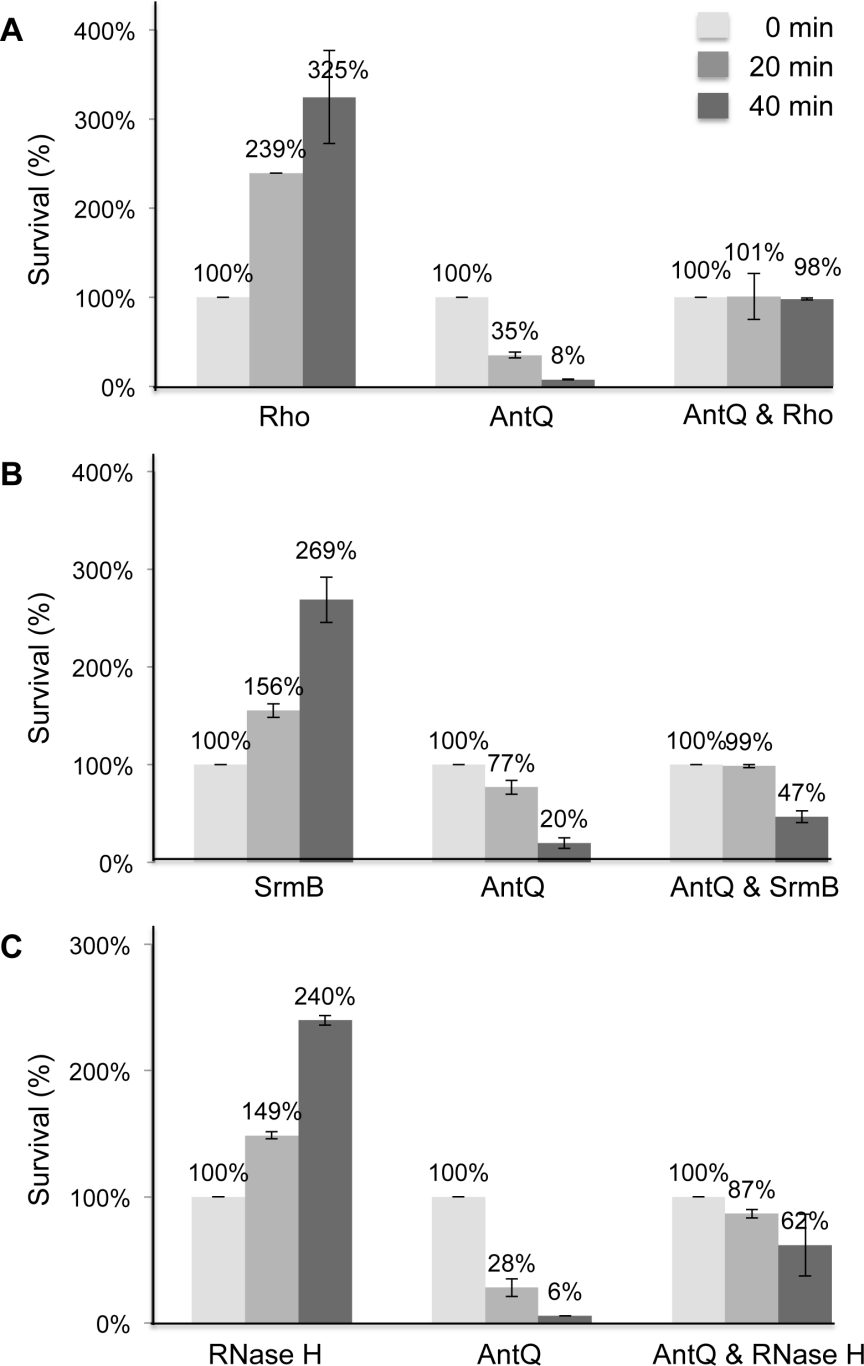
In *trans* expression of *rho*, *srmB* and *rnhA* rescues cells from AntQ toxicity. *Salmonella* cells were transformed with P*tac*-*antQ*-*lacI*, and either (A) P*rho*-*rho* (p15A origin), (B) PBAD-*srmB* (p15A origin) or (C) P*rnhA*-*rnhA* (p15A origin). In these constructs, Rho and RNase H are produced from their own promoters, whereas SrmB production was induced by arabinose at the time of dilution. IPTG (0.2 mM) to induce *antQ* was added 60 minutes after dilution. Samples were taken prior to the addition, 20 and 40 minutes after the addition of IPTG. Survival rates were calculated using the CFU of the first time point as the 100% reference.

We suspected that expression of *rho* and *srmB* increased in response to transcription elongation related stress. Thus, we examined whether co-expression of *antQ* with these genes would abolish the toxic effect of AntQ. Survival assays show that co-expressing *rho* with *antQ* help to rescue cells from AntQ-mediated toxicity. After 40 minutes of induction, survival of cells expressing *antQ* alone dropped to ~8% of their original amount, whereas cells expressing both *antQ* and *rho* managed to maintain a high CFU count, indicating that Rho can halt the toxicity inflicted by AntQ. Likewise, survival assays in which *srmB* and *antQ* were co-expressed demonstrated that SrmB prevented AntQ toxicity (Fig 8A and 8B). Together the results show that proteins that harbor RNA helicase activity impede the toxic effects of transcription anti-termination.

### Resolving R-loop by RNase H rescues cells from AntQ toxicity

Unregulated transcription elongation increases formation of DNA-RNA hybrids upstream of RNA polymerase (R-loops). The resulting R-loops may initiate DNA replication independently of *oriC*, leading to DNA damage [33, 34]. RNase H is an evolutionary conserved helicase that resolves R loops, thus protecting genomic DNA from breaks [30, 35]. Survival rates of cells co-expressing *antQ* and *rnhA* encoding RNase H were 10 fold higher than those expressing *antQ* alone, suggesting that the toxic effects of AntQ result from DNA damage due to the creation of R-loops (Fig 8C).

### *antQ* expression leads to genome condensation

**Fig 9:**
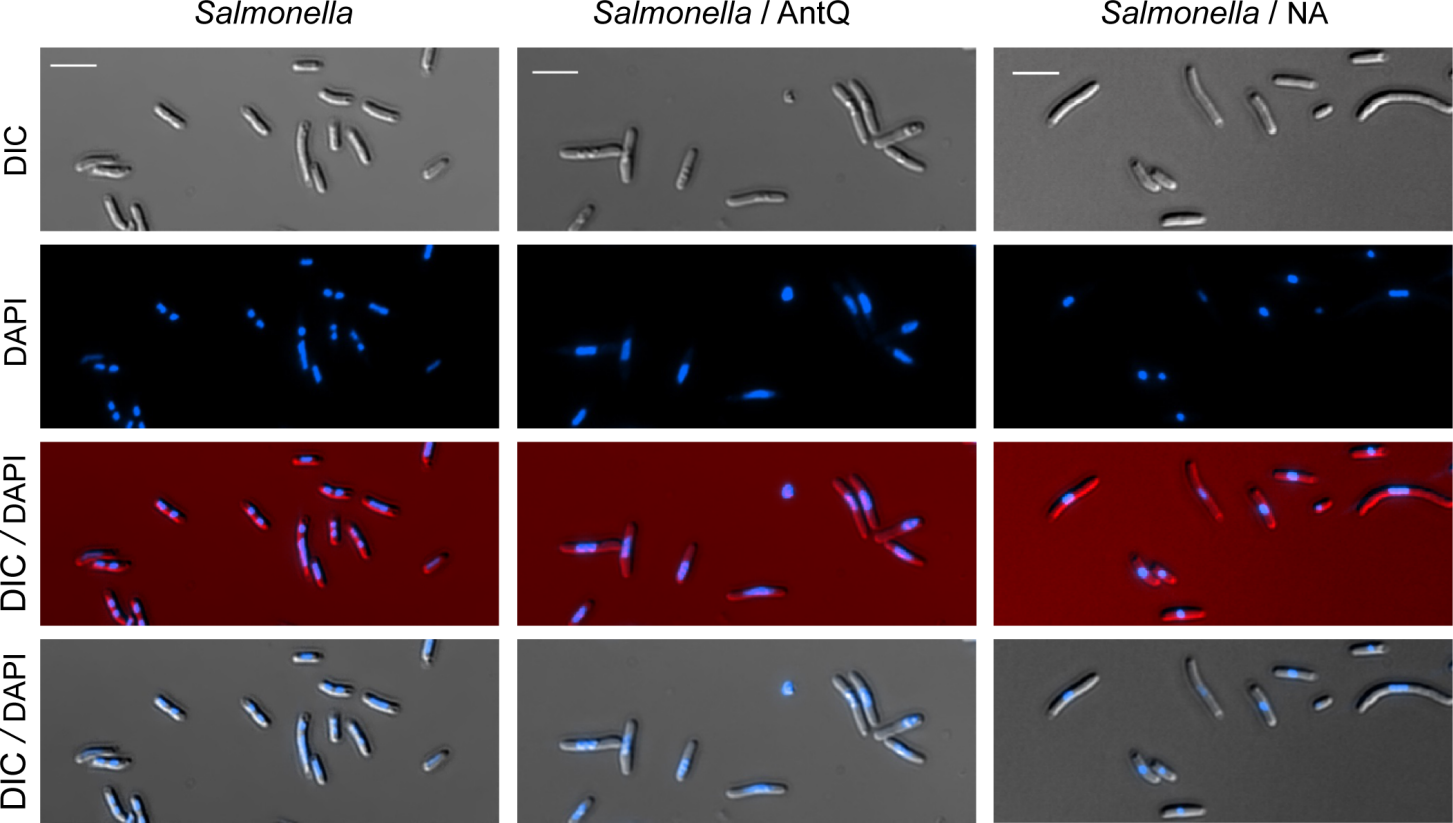
Expression of *antQ* in *Salmonella* results in genome condensation. Fluorescence microscopy images. Cultures of *Salmonella* carrying P*tac*-*lacI* or P*tac*-*antQ*-*lacI* were diluted 1/100 and let grown for 1 hour prior to the addition of 1 mM IPTG or 100 μg/ml nalidixic acid. Samples were taken at 60 min. of treatment. DNA stained blue with DAPI.

**Fig 10:**
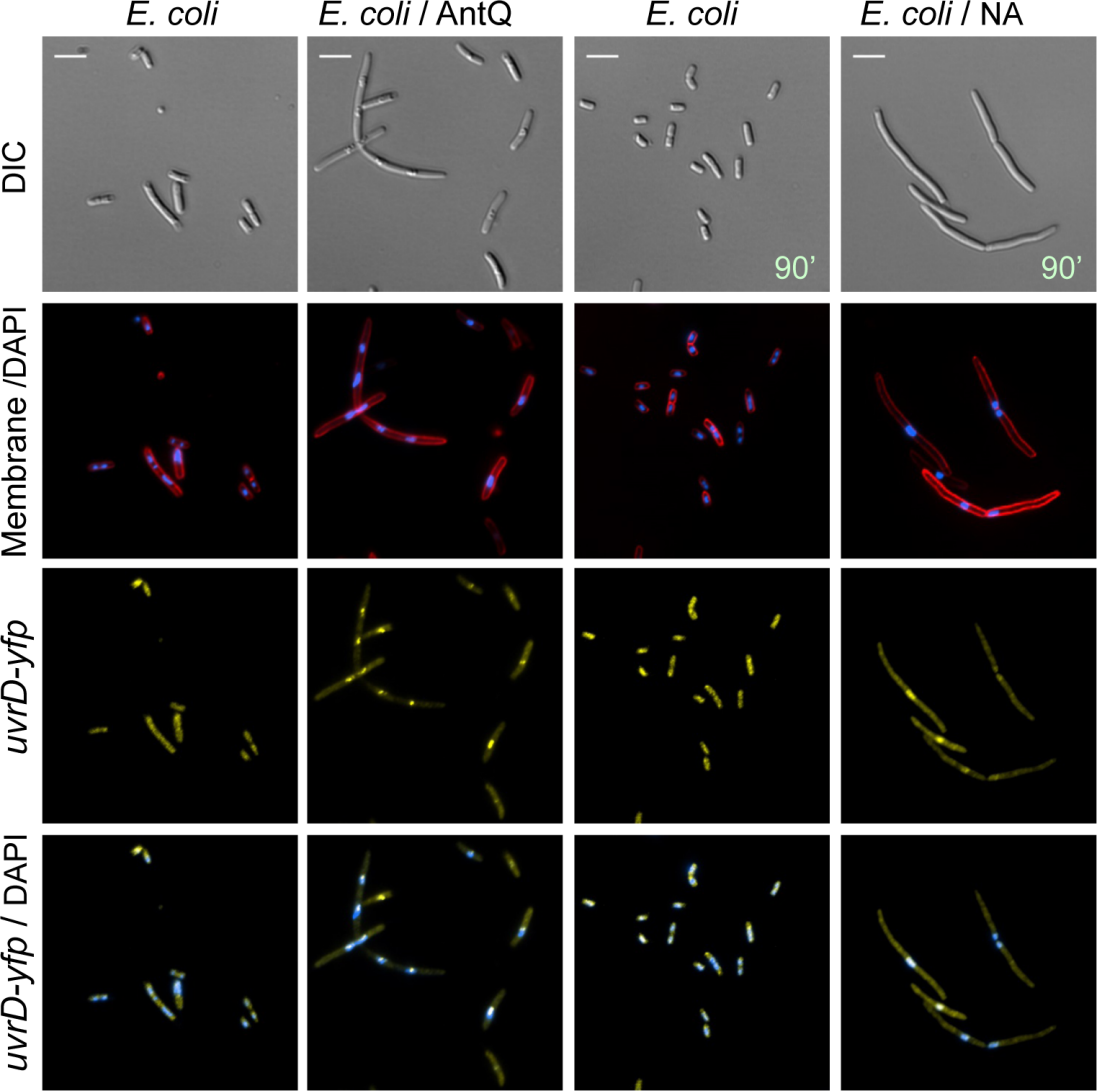
Expression of *antQ* in *E*. *coli* results in genome condensation. Fluorescence microscopy images. Cultures of *E*. *coli* (RW118) carrying *uvrD-yfp* fusion as a single copy in the native position in the chromosome and P*tac*-*lacI* or P*tac*-*antQ*-*lacI* were diluted 1/100 and let grown for 1 hour prior to the addition of 1 mM IPTG or 100 μg/ml nalidixic acid. Samples were taken at 60 and 90 min. of treatment, respectively. DNA stained blue with DAPI and cell membranes stained red with FM4-64.

It is well documented that exposure of bacteria to detrimental stressful conditions impairing protein synthesis or causing DNA damage, triggers genome condensation [36]. We visualized the effect of *antQ* expression on chromatin morphology by florescence microscopy. Images of *Salmonella* expressing a control plasmid display, as excepted, chromatin spread over the entire cytoplasm. In contrast, images of *Salmonella* expressing *antQ* reveal condensed chromatin morphology. Likewise, exposure of *Salmonella* cells to nalidixic acid (NA), a pleiotropic drug that inflicts diverse DNA lesions (nicks, gaps, and DSBs) [36] resulted in genome condensation (Fig 9). Furthermore, *E*. *coli* cells expressing *antQ* or exposed to NA exhibit genome condensation, similarly to *Salmonella*, indicating that *antQ* toxicity is mechanistically conserved. The *E*. *coli* UvrD protein is a DNA helicase/translocase that functions in methyl-directed mismatch repair (MMR) nucleotide excision repair (NER) and more broadly in genome integrity maintenance [37]. Recent studies in *E*. *coli* have shown that UvrD can act as an accessory replicative helicase that resolves conflicts between the replisome and transcription complexes [37–39]. Using *uvrD-yfp* [40], we demonstrate *in vivo* localization of the fluorescently tagged *uvrD* to the nucleoid upon expression of *antQ* and upon exposure to DNA damaging agents (Fig 10). Together our data show that the function of the phage antiterminator protein is wide-ranging causing changes in bacterial chromatin morphology.

## Discussion

### Dual regulatory function of IsrK small and messenger RNA

**Fig 11:**
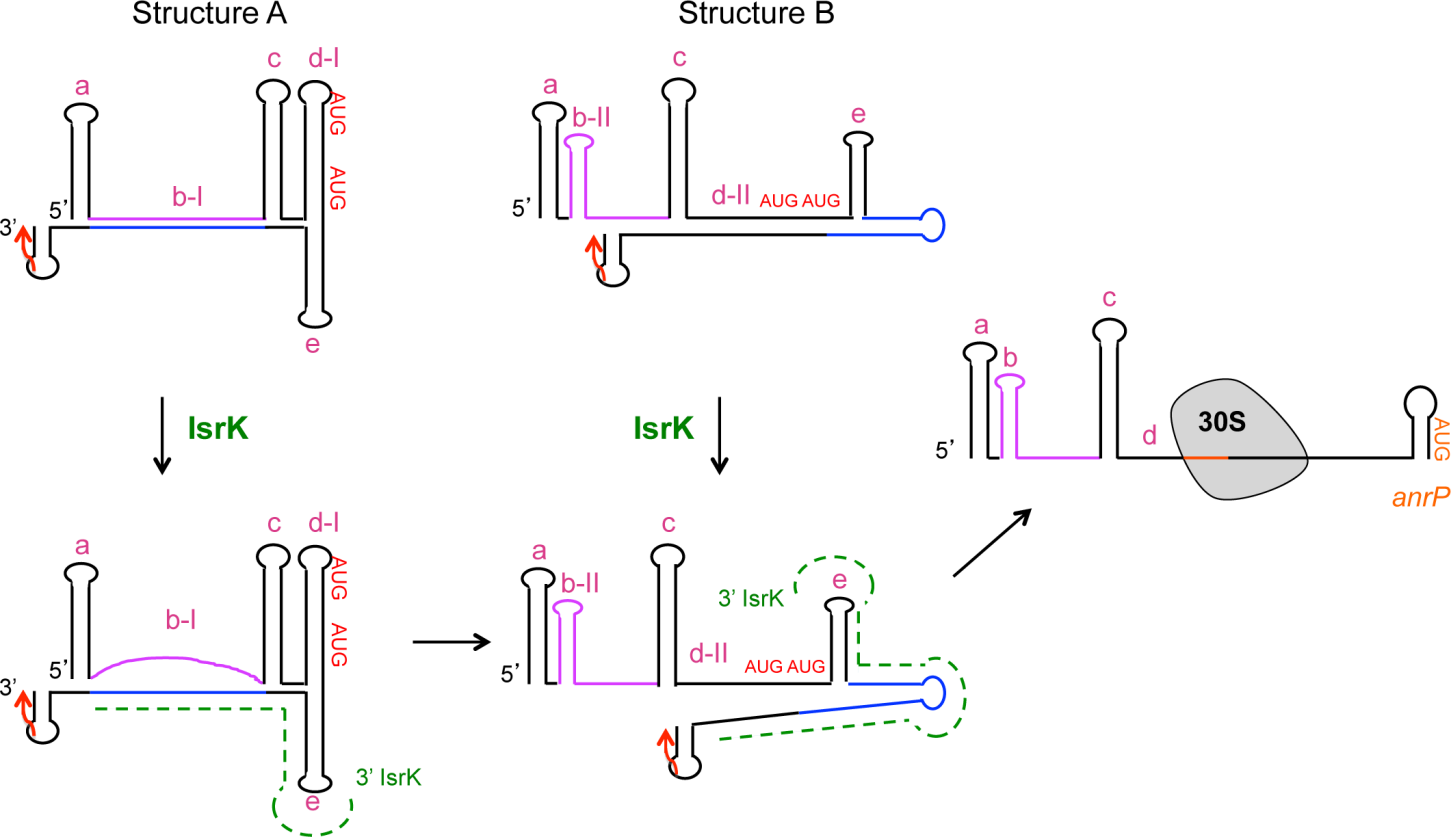
A model for *orf45-anrP* translation control by IsrK expressed in *trans*. The full-length wild type RNA forms a stable translationally inactive structure A. Some of the RNA forms structure B. IsrK sRNA expressed in *trans* binds the lower strand of helix b-I (blue), releasing the upper strand (purple) to form hairpin b-II, thus shifting the equilibrium towards structure B. In structure B, IsrK binding of the lower strand of helix d-II, renders the RBS of *orf45* accessible to ribosomes. Ribosomes bound to *orf45* elongate downstream to translate *anrP*. The two initiation codons and the stop of *orf45* are marked in red (AUG and arrow). IsrK sRNA is marked in green (dashed line).

In this study we show that a subset of the IsrK sRNA transcripts reads through its transcription terminator to form a translationally inactive bi-cistronic mRNA. Concomitantly, short IsrK RNA acts in *trans*, interacting with the inactive transcript to promote formation of a translationally active structure, in which *orf45* translation leads to *anrP* translation by translational coupling (model Fig 11). In bacteria, translational coupling provides a mechanism to coordinate expression of multiple proteins with adjacent or overlapping coding sequences. Ribosomes terminating translation of upstream ORF dissociate and re-initiate translation at the downstream RBS [41, 42]. Re-initiation is enabled due to ribosomes elongating along the upstream ORF to unfold mRNA structures that sequester the downstream ribosome-binding site. Such an example is PhrS sRNA that activates translation of *pqsR* mRNA by interaction with a sequence sequestering the RBS of an ORF upstream of *pqsR* [43]. We find that inserting a stop codon proximal to the translation initiation site of *orf45* reduces IsrK-controlled *anrP* translation activation, indicating that translational coupling is necessary for AnrP synthesis. Given that the structural changes caused by IsrK and/or ribosome binding at the *orf45* RBS do not seem to involve structural changes in the RBS of *anrP*, we suggest that the translational coupling between *orf45* and *anrP* requires ribosome elongation from the RBS of the *orf45* downstream to *anrP*.

A structural homolog of IsrK is SeqA RNA of P4-like phages [44]. In the lysogenic state P4 prevents expression of its own replication genes by premature transcription termination. The factor responsible for efficient termination is CI RNA that is generated by processing of a primary untranslated transcript. CI RNA acting as an antisense RNA leads to transcription termination by pairing with two complementary sequences, *seqA* and *seqC* located upstream and downstream of CI, respectively [45–47]. In *Salmonella*, transcriptome analysis revealed the existence of a stable non-coding RNA species downstream of IsrK (STnc1160) [8]. Our RNA analysis detected STnc1160 in wild type cells but not in a strain deleted of the *isrK* promoter, suggesting that STnc1160 is generated by processing of the readthrough transcript initiating from the *isrK* promoter (S3 Fig). It is possible that STnc1160 similarly to CI modulates transcription termination at IsrK Rho-independent terminator. Since IsrK and STnc1160 share complementary sequences, IsrK binding of STnc1160 renders it inactive as a termination factor leading to transcription readthrough and thus to grow arrest. However, in experiments of co-expression of *isrK* and *orf45* (STnc1160) in which STnc1160 was constitutively expressed, IsrK mediated growth arrest was even more pronounced (S14 Fig).

In S3B Fig, we present a northern blot showing the levels of chromosomally and plasmid encoded *isrK*. It is interesting to note that in addition to short IsrK, our analysis revealed a stable transcript (*isrK-orf45’)* that is generated by processing of the long polycistronic transcript. *isrK-orf45’* species is observed upon expression of plasmid-encoded *isrK* in wild type cells (lane 6), as well as upon expression of chromosomally-encoded *isrK* (see lane 1–3), indicating that the pattern detected with high level expression is valid with chromosomally-encoded *isrK*. In addition, the results demonstrating that Gifsy-1 phage induction by IsrK requires an intact *isrK* locus, whereas Gifsy-1 induction by AnrP is independent of *isrK* locus, further substantiate the regulatory cascade we present for the *isrK* locus and signify the biological relevance of this locus to phage activation. Moreover, we show that wild type cells grown in minimal medium to stationary phase exhibit prophage induction, whereas *isrK* promoter deletion mutant (ΔP*isrK*::*frt*) fails to produce phages (S15 Fig). These findings indicate that IsrK is an important player in initiating prophage induction. Concerning the conditions inducing *isrK* expression, we find that IsrK levels increase at stationary phase and under low Mg2+ conditions (S15 Fig). *Salmonella* global transcriptome analysis carried out by Kröger et al [8] shows that IsrK levels increase during conditions such as low Fe2+ shock, oxygen shock and growth in InSPI2 medium. In addition they find that the levels of the transcript encoding *orf45* resulting by transcription elongation through the *isrK* transcription terminator (STnc1160) increase during low Fe2+ shock, InSPI2 and late stationary phase. Together, the data indicate that *isrK* short and long forms are produced under a variety of environmental conditions.

The majority of the sRNA genes are encoded within intergenic regions acting in *trans* to control expression of physically unlinked target genes. However, it is now increasingly appreciated that in addition to intergenic regions, many sRNAs originate from the 5’ or 3’ regions of coding mRNAs. Such examples are 3’ UTR derived sRNAs that are generated either by internal processing of the related mRNA, as in the case of RybD or produced as a primary transcript like MicL and DapZ [7, 48]. Generated from within protein coding loci, these sRNAs act in *trans* controlling expression of unlinked target mRNAs. Likewise, SreA and SreB originate from 5’ UTRs of two S-adenosylmethionine (SAM) riboswitches, and base pair with the unlinked *prfA* mRNA to repress translation [49]. In *Staphylococcus aureus*, SprA1AS is transcribed from the strand opposite to SprA1 target mRNA encoding pepA1 ORF. The antisense RNA SprA1AS acts in *trans* by base pairing with the 5’ domain of SprA1 to repress pepA1 translation by occluding its RBS [50]. Somewhat different is the archaeal RNA regulator, sRNA162. sRNA162 masks the RBS of MM2441 by binding MM2440-MM2441 mRNA internally [51]. Biochemical studies demonstrated that in addition to in *trans* binding of MM2441 RBS, encoded opposite of MM2442, the 5’-end of sRNA162 targets the 5’-untranslated region of the cis-encoded MM2442 mRNA. However, the regulatory outcome of this interaction is as yet unknown.

The mechanism of expression regulation of the *isrK* locus is unique, representing the first example of an RNA that acts as a small RNA on its own mRNA. On the one hand, IsrK is part of a translationally inactive target mRNA, whereas on the other hand the short RNA species acts in *trans* to enable translation of the target mRNA. Therefore one mutation within IsrK RNA yields two different phenotypes; when located in the long target mRNA it increases translation whereas the short mutant IsrK RNA can no longer activate translation.

### AntQ modulates bacterial core genome transcription termination

Increasing evidences indicate that in prokaryotes and eukaryotes, common transcription-replication encounters lead to blockage of replication that is often accompanied by DNA damage and genome instability. In bacteria, because replication and transcription proceed simultaneously on the same template DNA, yet DNA replication forks move 10 to 30 times faster than do RNA polymerases, both co-directional, and head-on collisions appear to be unavoidable [34]. Transcription-replication conflicts may also result from stalled transcription elongation complexes, as they form stable barriers to the replication machinery. These complexes increase the production and/or the length of DNA-RNA hybrid structures within the transcription bubble, causing the region of complementary single-stranded DNA to loop out. The resulting R-loops may initiate DNA replication independently of *oriC*, leading to DNA damage [34]. We find that expression of the antiterminator protein results in bacterial growth arrest and ultimately cell death. Co-expression of *antQ* with transcription termination factor Rho rescues cells from the toxic effects of the antiterminator protein. Likewise, survival rates of cells co-expressing *antQ* and *rnhA* encoding RNase H were 10 fold higher than those expressing *antQ* alone. Given that RNase H protects genomic DNA from breaks by resolving R-loops, suggests that the toxic effects of AntQ result in part from DNA damage due to the creation of R-loops. In accordance, we find that expression of *antQ* affects bacterial chromatin morphology. Fluorescence microscopy images of *E*. *coli* and *Salmonella* expressing the antiterminator protein reveal condense chromatin morphology. Fluorescently tagged UvrD localizes to the nucleoid upon expression of the antiterminator protein as well as upon exposure to NA, a DNA-damaging agent. *uvrD* is a member of DNA helicase superfamily 1 and part of the SOS regulon. During SOS response the intracellular level of UvrD increases approximately three fold. UvrD functions in methyl-directed mismatch repair (MMR) nucleotide excision repair (NER) and genome integrity maintenance. Recent studies have demonstrated that UvrD contributes to genomic integrity by resolving conflicts between transcription and DNA repair complexes [37–39]. UvrD binds RNA polymerase in blocked transcription elongation complexes, forcing it to slide backwards along the DNA. This backwards sliding exposes DNA lesions that are out of reach allowing the nucleotides excision repair enzyme to access the site of damage.

A question remains, are the massive effects on chromatin structure detected upon IsrK/AntQ overproduction physiologically relevant? Quantitation analysis of *antQ* mRNA levels indicates that at around two hours upon exposure to in *trans* IsrK, during the time that the long transcript *isrK-orf45-anrP* is detected, the copy number of *antQ* is increased by 6 fold (2.5x10-2 per 16S rRNA). During the first nine minutes of exposure to hydrogen peroxide the copy number of *antQ* increases by 4 fold (1.2x10-2 per 16S rRNA). Thus, it is conceivable that the phenotype observed with overproduction of *antQ* is biologically relevant. Regardless, the toxic phenotype of *antQ* is intriguing, a phage encoded antiterminator protein known to facilitate transcription antitermination at a specific phage terminator, is in fact, wide-ranging capable of affecting bacterial core genome sites. Given the morphological appearance of cells expressing the antiterminator protein and that the toxic phenotype is reversed by opposing functions, including transcription termination and elimination of R loop, AntQ, by affecting transcription elongation causes DNA damage. Whether the phage exploits *antQ* to modulate vital bacterial machineries or bacterial cells use the Q-like antiterminator core genome native sites for self-inhibition upon phage infection remains to be addressed in further studies.

## Materials and Methods

### Bacterial growth conditions

*Salmonella Typhimurium* SL1344 cells were grown at 37°C (200 rpm) in LB medium (pH 6.8). Ampicillin (100 μg/ml), Chloramphenicol (20 μg/ml) and kanamycin (40 μg/ml) were added where appropriate. Induction of PBAD promoter was obtained with arabinose (0.2%), whereas P*tac* promoter was induced with IPTG as indicated. (List of strains, plasmids and DNA primers used in this study appear in S2–S4 Tables)

### Strain construction

Gene deletion mutants were generated using the gene disruption method as described [52]. For construction of deletion mutants, chloramphenicol or kanamycin cassettes were amplified from plasmids pKD3 and pKD4, respectively [53]. The PCR product (5–10 μg) purified using the Wizard SV PCR clean-up system (Promega, Madison, WI) was introduced into arabinose treated LB5010 cells [54] carrying pKD46 cells [52] and chloramphenicol or kanamycin-resistant colonies were selected. The deletion mutation was transferred into a wild type SL1344 genetic background by transduction using the P22 bacteriophage. The resistance gene was eliminated using pCP20 [53]. In Δ*anrP*::*kan*, the chromosomal region flanked by genome coordinates 2759753 and 2760316 (GenBank entry CBW18679.1) was replaced by the *kan* gene using primers 1571 and 1572. *anrP* gene disruption was examined by PCR using flanking primers, 1528 and 1529. In Δ*antQ*::*cat*, the chromosomal region flanked by genome coordinates 2760589 and 2761266 (GenBank entry CBW18680.1) was replaced by the *cat* gene using primers 1573 and 1458. *antQ* gene disruption was examined by PCR using flanking primers, 1542 and 1486. In Δ(SL2575-SL2576)::*cat* and Δ(SL2575-SL2576)::*kan* the chromosomal region flanked by genome coordinates 2756646 and 2757568 (GenBank entry CBW18677.1 and CBW18676.1) was replaced by the *cat* gene using primers 1615 and 1616, or *kan* cassette using primers 2149 and 2150. (SL2575-SL2576) gene disruption was examined by PCR using flanking primers, 1617 and 1618. In SL1344 *antQ*::*cat* Δ(SL2575-SL2576)::*kan* double deficient mutant, a P22 lysate generated from Δ*antQ*::*cat* was transferred into Δ(SL2575-SL2576)::*kan* by transduction and the resistant genes were later eliminated using pCP20 to generate a mutant deficient of the entire region SL1344 *antQ* to (SL2575-SL2576)::*frt*. The disruption was examined by PCR using flanking primers, 1761 and 1618. To construct strains carrying SPA tags in the chromosome; SL1344 *orf45*-SPA-*kan* and SL1344 *anrP*-SPA-*kan*, primers were designed to amplify the sequential peptide affinity (SPA) tag together with the kanamycin resistance cassette from the plasmid pJL148, and flanked by 45 nt of sequence homologous to the insertion region, as described before. SL1344 *orf45*-SPA-*kan* and SL1344 *anrP*-SPA-*kan* were constructed using primers 2122, 2123 and 2209, 2210 respectively. The PCR products were purified from gels and used to transform SL1344 cells carrying pKD46 plasmid [52]. Insertions were confirmed by sequencing of PCR products generated using primers 1987, 2227 (198 nt of *orf45*-SPA-*kan*) and 839, 2227 (196 nt of *anrP*-SPA-*kan*). The products were sequenced using primer 2227. *uvrD-yfp* fusion [40] was transferred into strain RW118 [55] by P1 transduction.

### Plasmid construction

To construct P*tac*-*isrK* and PBAD-*isrK*, *isrK* sequence from its transcription start site plus 37 nt downstream of its transcription terminator was PCR amplified from SL1344 chromosomal DNA using primers 1364 and 1365 and cloned into the EcoRI and HindIII restriction sites of pRI and pJO244 respectively. To construct P*tac*-*antQ*-*lacI*, *antQ* sequence from its ATG plus 22 nt downstream of its stop codon was PCR amplified from SL1344 chromosomal DNA using primers 1544 and 1510 and cloned into the EcoRI and SalI sites of pKK177-3-*lacI*. In this plasmid, *antQ* translation is directed by an artificial translation initiation signal found in the right position in pKK177-3-*lacI*. To construct P*tac*-*anrP*-*lacI*, *anrP* sequence from its ATG plus 40 nt downstream of the stop codon was PCR amplified from SL1344 chromosomal DNA using primers 1893 and 1897 and cloned into the EcoRI and PstI sites of pKK177-3-*lacI*. In this plasmid, *anrP* translation is directed by an artificial translation initiation signal found in the right position in pKK177-3-*lacI*. To construct P*rho*-*rho* (p15A origin), a DNA fragment including both the promoter of Rho and the *rho* gene (-200 to 112 nt downstream of the stop codon) was PCR amplified from SL1344 chromosomal DNA using primers 1872 and 1862 and cloned into the BglII and HindIII sites of pACYC184. To construct PBAD-*srmB* (p15A origin), a DNA fragment containing *srmB* from nucleotide146 upstream of the ATG to nucleotide 2 downstream of the stop codon was PCR amplified from SL1344 chromosomal DNA using the primers 1884 and 1885 and cloned into the PstI and HindIII sites of pEF21. In this plasmid, *srmB* translation is directed by its own translation initiation signal. To construct P*rnhA*-*rnhA* (p15A origin), a DNA fragment including both the promoter of *rnhA* and the *rnhA* gene (-112 to 89 nt downstream of the stop codon) was PCR amplified from SL1344 chromosomal DNA using primers 1907 and 1908 and cloned into the XbaI and BamHI sites of pACYC184. To construct *lacZ* fusions in single copy plasmids (pBOG551 and pBOG552), the origin of replication of pRS551 and pRS552 [56] was replaced by the origin of replication of pZS*24 (pSC101*) [57]. The origin of replication was amplified using primers 2032 and 2042 and cloned into the PstI and SalI sites of pRS551 and pRS552 plasmids. To construct wild type fusions PCR fragments carrying P*isrK-orf45-anrP* were amplified from genomic DNA using primers 1512 and 1703, digested and cloned into the EcoRI and BamHI sites of pGEM3. The P*isrK-orf45-anrP* fragment was sub-cloned into pBOG551 and pBOG552 using the EcoRI and BamHI sites. All fusion mutants were constructed by transferring mutated P*isrK-orf45-anrP* fragments from pGEM3 into pBOG plasmids, as described above. To construct PL*tetO-1-orf45* (p15A origin), we first deleted the luc gene of pZA31-*luc* by PCR using primers 1989 (KpnI) and 1990 (phosphorylated). Thereafter, *orf45* sequence from its second ATG to its stop codon was PCR amplified from SL1344 chromosomal DNA using primers 1987 (KpnI) and 1988 (phosphorylated). The two PCR fragments (orf45 and pZA31) where then ligated.

### Random mutagenesis

To carry out random mutagenesis, 1 volume (1.5 μg) of plasmid DNA carrying P*isrK-orf45-anrP-'lacZ* (pSA81) was mixed with 5 volumes of phosphate solution (0.5 M NaH2PO4, 1 mM EDTA, adjusted to pH 6 with NaOH), and 4 volumes of hydroxylamine solution (1 M hydroxylamine hydrochloride (Fluka) in phosphate solution, adjusted to pH 6 with NaOH). The mixture was incubated at 65°C for 2 hours, then dialyzed overnight against TE buffer (10 mM Tris-HCl at pH 8, 1 mM EDTA) at 4°C and again for 2.5 hours. The mutagenized plasmid was used to transform MC4100 cells. Dark blue colonies were picked from LB plates containing 40 μg/ml 5-bromo-4- chloro-3-indolyl-β-D-galactopyranoside (x-gal) (Inalco). Plasmid inserts from selected colonies were sequenced [56].

### Site directed mutagenesis

Mutations A107C, AU119-120UA, G28A, C162U, G31A, C159U, G114A, G173A and C175U were generated by PCR using plasmid carrying P*isrK-orf45-anrP* (pSA77) and two tail-to-tail divergent primers of which one carried the desired mutation. The PCR product was gel purified, subjected to blunt end ligation and the mutated plasmid was digested with EcoRI and BamHI for sub-cloning to pBOG551 and pBOG552. The double mutants A107C/G121A, G28A/C162U, G31A/C159U and G114A/C175U were constructed using P*isrK-orf45*A107C*-anrP* (pSA77A107C), P*isrK*G28A*-orf45-anrP* (pSA77G28A), P*isrK*G31A*-orf45-anrP* (pSA77G31A) and P*isrK-orf45*G114A*-anrP* (pSA77G114A), respectively as template and two tail-to-tail divergent primers of which one carried the desired second mutation. The PCR product was gel purified, subjected to blunt end ligation and the mutated plasmid was digested with EcoRI and BamHI for sub-cloning to pBOG551 and pBOG552 [56].

### Survival assay

Overnight cultures of *S*. *typhimurium* SL1344 or SL1344 carrying plasmids were grown from fresh transformation plates. Each strain was grown in duplicates. Starters were diluted 1/100 in 15 ml LB (125 ml Erlenmeyer flasks) and grown at 37°C (200 rpm). Arabinose (0.2%) was added at the time of dilution where indicated. One hour after dilution, IPTG was added to a final concentration of 0.2 mM to induce expression of *antQ*. Samples were taken prior to, 20 and 40 minutes after the addition of IPTG, diluted in 1X PBS and plated. Each sample was plated twice. Colonies were counted and percentage of survival rate was calculated.

### β-galactosidase assays

Overnight cultures were diluted 1/100 in 20 ml LB medium supplemented with ampicillin and kanamycin, and grown to OD600 ~ 1.0. To induce IsrK, arabinose (0.2%) was added at the time of dilution. β-galactosidase activity was assayed as described [58].

### Phage induction

To detect Gifsy-1 phage induction by AnrP, overnight cultures of *S*. *typhimurium* wild type, *isrK* promoter deletion mutant (ΔP*isrK*::*frt*), and Gifsy-1 lysis proteins deletion mutant ΔSL2575-SL2576::*frt* carrying P*tac*-*lacI* or P*tac*-*anrP*-*lacI* were diluted (1/100) in LB medium supplemented with 10 mM MgSO_4_ and ampicillin and grown at 37°C to OD600 ~ 0.3. Thereafter, IPTG (0.2 mM) was added to induce expression of *anrP*. At 2 hr after induction the cultures were treated with chloroform to release phage particles. 5μ of the supernatant were plated on LT2 (lambda sensitive) bacterial lawn made with soft agar. To detect Gifsy-1 phage induction by IsrK, wild type and ΔP*isrK*::*frt* mutant carrying PBAD and PBAD-*isrK* were diluted, grown and their phages plated as above. Arabinose (0.2%) was added at the time of dilution for induction of IsrK. To detect oxidative stress dependent phage induction [15, 59] H2O2 (0.1 and 0.5 mM) was added at OD600 ~ 0.3 and phages were plated as above.

### Growth conditions of real time-PCR experiments

To measure the effect of AnrP on expression at *antQ* locus, ΔSL2575-SL2576::*frt* cells carrying P*tac*-*lacI* or P*tac*-*anrP*-*lacI* were grown to OD600 ~ 0.5. Total RNA was extracted prior to and at 25 min of induction with IPTG (1 mM). To measure the effect of IsrK on expression at *antQ* locus, wild type cultures carrying PBAD and PBAD-*isrK* (treated with arabinose (0.2%) at the time of dilution) were grown and total RNA was extracted at time points as indicated. To measure expression at *antQ* locus upon phage induction, H2O2 (0.1 mM) was added at OD600 ~ 0.3. Total RNA was extracted prior to and upon exposure to H2O2 (as indicated) and cDNA was prepared for real time PCR. To monitor expression of chromosomally encoded-*isrK*, wild type and (ΔP*isrK*::*frt*) mutant strains were grown in LB medium to OD600 ~ 0.3, 0.6, 1.0 and for 8 hours or in low MgCl_2_ N-minimal medium to OD600 ~ 0.3 [6].

### RNA extraction

Overnight cultures of *S*. *typhimurium* SL1344 or SL1344 carrying plasmids were diluted and grown as described before. To isolate total RNA, the cultures were pelleted and re-suspended in 50 μl 10 mM Tris–HCl (pH 8) containing 1 mM EDTA. Lysozyme was added to 0.9 mg/ml and the samples were subjected to three freeze-thaw cycles. Total RNA was purified using TRI reagent (Sigma) according to the manufacturer’s protocol.

### Real time PCR

RNA concentrations were determined using a NanoDrop machine (NanoDrop Technologies). DNA was removed by DNase treatment according to the manufacturer’s instructions (RQ1 RNase free DNase, Promega). About 1 μg DNA-free total RNA was used for cDNA synthesis using MMLV reverse transcriptase and random primers (Promega). Quantification of cDNA was performed by real-time PCR using SYBR-green mix (Absolute SYBR GREEN ROX MIX, ABgene) with Rotor gene 3000A (Corbett) according to manufacturer’s instructions. Specific primer pairs were designed according to the Guidelines for Amplicon and Primer Design (http://www.tamar.co.il/tamar-laboratory-supllies/guidelines-amplicon-primer-design/). The level of 16S rRNA (*rrsA*) was used to normalize the expression data for each target gene. The relative amount of cDNA was calculated using the standard curve method. A standard curve was obtained from PCR on serially diluted genomic DNA as templates and was analyzed using Rotor-gene analysis software 6.0.

### SDS-PAGE analysis of total protein

Overnight cultures of wild type cells carrying a control plasmid (P*tac*) or an AntQ expressing plasmid were diluted 1/100 and grown at 37°C. IPTG (1 mM) was added at OD600 ~ 0.2 for AntQ induction. Samples were taken 30 and 60 minutes after exposure to IPTG, pelleted and then fluidized in 1X Laemmli sample buffer, heated at 95°C for 5 min and centrifuged for 5 min. 20 μl of each sample were analyzed by 12% SDS-PAGE (9 mA, 1X running buffer for 24 hours at 4°C). To visualize the proteins, the gel was stained for 30 minutes at 37°C (coomassie blue staining). The mass spec data of the band (see S13 Fig) were analyzed based on coverage, which represents the percentage of the protein that was sequenced; area, which describes the fraction of the specific protein out of the sample, and the number of unique peptides, found to only match this specific protein. The high scored proteins were considered for further analysis based on their relevant function in transcription elongation or termination.

### Western of SPA tagged *orf45* and *anrP*

Overnight cultures of SPA-tagged strains carrying control plasmid (PBAD) or IsrK expressing plasmid (PBAD–IsrK) were diluted 1/100 and grown shaking at 37°C. Arabinose (0.2%) was added at the time of dilution. Samples were taken at indicated OD600, pelleted and then fluidized in 1X Laemmli sample buffer, heated at 95°c for 5 min and centrifuged for 5 min. Samples of 3x107 cells were analyzed on SDS-PAGE (12% and 15% for ORF45-SPA and AnrP-SPA, respectively) [60]. The proteins were transferred to a nitrocellulose membrane (Invitrogen), the blots were blocked with skim milk (2.5% for 1 hour) and probed with FLAG M2-AP monoclonal antibody (Sigma-Aldrich) according to the manufacturer’s protocol. The tagged proteins were visualized using secondary antibody Anti-Mouse IgG-Alkaline Phosphatase (Sigma-Aldrich) based on Alkaline Phosphatase development protocol.

### *In vitro* RNA synthesis

The RNAs; *isrK-orf45* wild type (from transcription start site to nucleotide 217 within *orf45* or to nucleotide 785 at the end of *anrP*) and mutants: *isrK*G31A-*orf45* (217 nt), *isrK*G31A-*orf45*C159U (217nt), *isrK*G28A-*orf45* (217nt), *isrK*G28A-*orf45*C162U (217nt) as well as IsrK sRNA (90 nt) wild type and G31A mutant were synthesized with phage T7 RNA polymerase (25 units; New England Biolabs) in 50 μl reactions containing 40 mM Tris-HCl (pH 7.9), 6 mM MgCl_2_, 10 mM diothiothreitol (DTT), 20 units RNase inhibitor (CHIMERx), 500 μM of each NTP and 200 ng of purified PCR templates carrying the sequence of the T7 RNA polymerase promoter. Synthesis was allowed to proceed for 2 hours at 37°C, and was terminated by phenol/chloroform extraction and ethanol precipitation in the presence of 0.3M ammonium acetate.

### Northern analysis

RNA samples (20 μg for detection of *isrK* and 30 μg for detection of STnc1160 and *orf45*) were denatured for 5 min at 65°C in 98% formamide loading buffer, separated on 8 M urea-6% polyacrylamide gels and transferred to Zeta Probe GT membranes (Bio-Rad Laboratories) by electroblotting. To detect IsrK RNA, the membranes were hybridized with [32P]-end-labeled *isrK* primer (1197) in modified CHURCH buffer [6]. STnc1160 and *orf45* were detected using anti-STnc4100 labeled riboprobe synthesized using PCR template (2316 and 2317) as previously described [6]. Riboprobe hybridization buffer contained 50% formamide, 3.5% SDS, 250 mM NaCl, 82 mM Na2HPO4, 40 mM NaH2PO4 at pH 7.2. After 2 hours at 50°C, the membranes were treated for 20 min at 50°C in 2X SSC, 1% SDS, 20 min at 55°C in 1X SSC, 0.5% SDS and 20 min at 60°C in 0.5X SSC, 0.1% SDS. To detect *isrK-orf45-anrP* full length RNA, samples (20 μg) were denatured for 10 min at 65°C in MOPS loading buffer, separated on 1.2% agarose gels and transferred to Zeta Probe GT membranes by capillary transfer [6]. The membranes were hybridized in modified CHURCH buffer using end-labeled *isrK* (1197) or *isrJ* (1471) specific primers.

### Native gels

To detect binding of IsrK to its templates *in vitro* synthesized RNAs wild type *isrK-orf45* and mutants (217 nt, 0.2 pmol) were incubated in 10 μl of Native Buffer (6.7 mM Tris-acetate (pH 7.4), 3.3 mM Na-acetate, 1 mM DTT and 10 mM MgCl_2_) for 3 minutes at 70°C and chilled on ice. Thereafter, *in vitro* synthesized IsrK or IsrKG31A RNAs were added as indicated in the Fig and incubated for 15 minutes at 37°C. The RNA samples were analyzed on 5% non-denaturing polyacrylamide gels (19:1) run at 50 volts in 20 mM Tris-HCl (pH 7.5), 60 mM KCl and 10 mM MgCl_2_ for 5–6 hours at 4°C as described before [56]. After its transfer to nylon membrane by electroblotting, the RNA was detected by probing with end-labeled *orf45* specific primer (1948). To detect RNA conformations the template RNAs wild type and mutants (217 nt, 0.2 pmol) were incubated in Native Buffer (as above) for 15 minutes at 37°C and then analyzed as describe above. The RNAs were also analyzed on denatured gels (see Northern analysis) using end-labeled *orf45* specific primer that detects *isrK-orf45* templates (1948).

### DMS footprinting of ribosome

Template RNA synthesized *in vitro* (0.7–1 pmol) was incubated in 50 mM Na-cacodylate (pH 7.4), 10 mM magnesium acetate, 100 mM NH4Cl and 2.5 mM β-mercaptoethanol for 3 minutes at 70°C and chilled on ice for 10 min. Thereafter, *in vitro* synthesized wild type IsrK or IsrKG31A (7–10 pmol) was added and the mixture was incubated for 15 minutes at 37°C. Pre-activated (30 minutes at 37°C) 30S ribosomal subunits (1.2–2.4 pmol) were added for 5 minutes prior to the addition of uncharged fMet-tRNA (12 pmol). The binding reactions were incubated for 15 min before DMS (0.5 μl, diluted 1:10 in ethanol) was added. The modification reaction was allowed to proceed for 5 minutes. Reactions were stopped with phenol/chloroform and precipitated with ethanol in the presence of 0.3 M sodium acetate, 1 μl of Quick-Precip (Edge BioSystems) and 20 μg yeast RNA. The modification sites were detected by primer extension using MMLV reverse transcriptase (Promega) and end-labeled primer (1948).

### Fluorescence microscopy

Fluorescence microscopy was carried out as described previously [61]. In brief, 1–2 ml cells were centrifuged, washed with 1X phosphate buffered saline (PBS) and finally re-suspended in 10–100 μl of PBS. The membrane was stained with FM4-64 (Molecular Probes, Invitrogen) at a final concentration of 10 μM or 1 μg/ml, respectively. DNA was stained with DAPI (Sigma-Aldrich) at a final concentration of 2 μg/ml. Cells were washed twice before microscopic examination. Cells were visualized and photographed using Nikon Eclipse Ti-E inverted microscope equipped with Perfect Focus System (PFS) and ORCA Flash 4 camera (Hamamatsu photonics). Images were processed using NIS Elements-AR software.

## Supporting Information

S1 Fig IsrK is toxic in cells deleted for Gifsy-1 phage lysis genes. *Salmonella* wild type and cells deleted of SL2575 and SL2576 genes encoding proteins of phage lysis and phage lysozyme superfamily, respectively, were transformed with P*tac* plasmid and P*tac-isrK*, expressing IsrK constitutively.
(PNG)

S2 Fig AntQ is detrimental. *Salmonella*, wild type, Δ*antQ*::*frt* and Δ*anrP*::*frt* were transformed with P*tac* plasmid and P*tac-isrK* expressing IsrK constitutively (A). (B) *Salmonella* deleted of the entire locus between *antQ* and the Gifsy-1 lysis genes SL2575-SL2576 (including) was transformed with plasmids expressing *anrP* or *antQ* from P*tac* promoter under the control of LacI repressor (pKK177-3-*lacI*). The transformations were plated on LB plates supplemented with IPTG (0.05 mM) and antibiotics. Plasmids are denoted in red.
(PNG)

S3 Fig IsrK sRNA and full-length polycistronic transcript at the *isrK* locus. Northern blot of RNA extracted from wild type and cells deleted of the *isrK* promoter in the chromosome (ΔP*isrK*) as well as cells carrying a control plasmid (PBAD) or an *isrK* expressing plasmid (PBAD-*isrK*) as indicated. Cultures carrying plasmids were induced with arabinose at dilution. (A) 1% agarose formaldehyde gel detecting the full-length transcript *isrK-orf45-anrP-isrJ*. The membrane was probed with 5’-end labeled IsrK and IsrJ specific primers. (B) 6% urea-PAGE detecting short IsrK (77nt) both chromosomally (lanes 1–3) and plasmid encoded (lanes 4–9). It is interesting to note that the northern also detects a stable transcript (*isrK-orf45’)* that was generated by processing of the long polycistronic transcript, (lanes 1–3 and 6). The membrane was probed with end-labeled *isrK* specific primer. The left panel (lanes 1–3) was exposed longer (3x) than the right panel. (C) 6% urea-PAGE carrying RNA extracted from cultures of wild type and ΔP*isrK* grown for 3 (OD600 of 1.0), 8 and 11 hours as indicated. The membrane was probed with fully labeled antisense of *orf45’*. The riboprobe detects two RNA species that are generated by processing of the read-through transcript; a ~180 nt long RNA (*isrk-orf45’*, also detected by *isrK* primer in (A) and a small species of ~ 80 nt that is generated by further processing of *isrk-orf45’*. The truncated RNA species (*orf45’*) was also detected in *Salmonella’s* transcriptome (see Discussion STnc1160 [8]). (D) Oxidative stress induces expression of *isrK*. Both short *isrK* and *isrK-orf45’* that is generated by processing of the long polycistronic transcript are detected in response to exposure to hydrogen peroxide (see Materials and Methods for details). The membrane was probed with end-labeled *isrK* specific primer. 5S RNA serves as a loading control.
(PNG)

S4 Fig High-levels of IsrK or AnrP lead to an increase in expression of *antQ* operon. Real-Time PCR of SL2581 mRNA detected in the presence of high levels of IsrK (PBAD-*isrK*) (A) or AnrP (P*tac*-*anrP*-*lacI*) (B). SL2581 is the second gene in *antQ* operon [8] that includes SL2582, SL2581, SL2580 and SL2579 (here denoted *antQ*). *Salmonella* carrying control, IsrK, and AnrP expressing plasmids were exposed to arabinose and IPTG to activate PBAD and P*tac* promoters, respectively (see also Materials and Methods). Two samples per treatment and two reactions per sample were analyzed.
(PNG)

S5 Fig Gifsy-1 phage induction by IsrK and AnrP. (A) Gifsy-1 phage induction by IsrK requires an intact *isrK* locus. Cultures of *S*. *typhimurium* wild type and *isrK* promoter deletion mutant (ΔP*isrK*::*frt*) carrying PBAD and PBAD-*isrK* were grown with arabinose to induce *isrK* expression for two hours. Thereafter, their phages were released by chloroform and plated on LT2 (lambda sensitive) as described in Materials and Methods. (B) Gifsy-1 phage induction by AnrP is independent of *isrK* locus. Wild type, (ΔP*isrK*::*frt*), and Gifsy-1 lysis proteins deletion mutant (ΔSL2575-SL2576::*frt*) carrying P*tac*-*anrP*-*lacI* were grown with IPTG to induce expression of *anrP* and their phages were collected and plated on LT2 (lambda sensitive) as described in Materials and Methods. (C) Oxidative stress dependent phage induction. H2O2 (0.1 and 0.5 mM) was added at OD600 ~ 0.3 and phages were plated as above.
(PNG)

S6 Fig Prophage induction leads to an increase in expression of *antQ* operon. Real-Time PCR of *antQ* and SL2581 mRNAs expressed upon phage induction by hydrogen peroxide as indicated in the Fig. Samples were taken and assayed as described in Materials and Methods. SL2581 is the second gene in *antQ* operon [8] that includes SL2582, SL2581, SL2580 and SL2579 (here denoted *antQ*). Two samples per treatment and two reactions per sample were analyzed.
(PNG)

S7 Fig Sequence conservation of the *isrK* locus including *isrK*, *orf45* and *anrP*. Representative genera from the original analysis (blast) are shown. Initiation and stop codons of *orf45* and *anrP* are marked in red. Complementary nucleotides between *isrK* and *orf45* are marked in green. The black arrows under the green sequences denote the position and the orientation of basepairing.
(PDF)

S8 Fig Amino acid conservation of ORF45 and AnrP. The signs represents full identity (*) conservation (.) and semi conservation (:). Red asterisks represent stop codons. In S. *dysenteriae orf45* seem to be fused to *anrP* (287 amino acids).
(PDF)

S9 Fig Binding of *isrK-orf45* by IsrK. RNAs (0.2 pmol), wild type *isrK-orf45* and mutant *isrK*G31A-*orf45* were incubated for 15 minutes at 37°C in the presence of increasing amounts of IsrK, as indicated. The samples were separated on non-denaturing polyacrylamide gels. Arrows indicate the two conformations observed. The target-RNAs were detected using an *orf45* specific labeled primer (1948). Analysis of the RNA samples on denaturing gels exhibits one form (see Fig 6C).
(PNG)

S10 Fig IsrK mutants G28A and G31A are not toxic in wild type cells. (A) Growth curves of wild type cells carrying control (PBAD) or *isrK* expressing plasmids; wild type, *isrK*G28A and *isrK*G31. (B) Northern blot comparing RNA levels of wild type and *isrK* mutants. RNA was extracted from cells deleted of the *isrK* locus (Δ*isrK* to *isrJ*) carrying plasmids as in A. Cells were induced with arabinose (0.2%) at the time of dilution and RNA was extracted at OD600 1.0
(PNG)

S11 Fig 30S binding at *orf45*-RBS is facilitated by wild type IsrK but not by IsrKG31A. *In vitro* synthesized RNA templates were incubated with and without 30S ribosomes, IsrK RNA or IsrKG31A prior to the addition of DMS. Thereafter, the samples were treated with phenol as described in Materials and Methods. The modified sites were detected by primer extension. In this experiment, we used increased concentrations of 30S ribosomal subunits (2.4 pmol). Under these conditions, primer extension termination sites can be detected at the marked G residues in the absence of DMS (lane 1). The authentic DMS modification sites at AAA residues are marked by red dots.
(PNG)

S12 Fig AntQ toxicity is not correlated with phage DNA. Growth curves (A) showing the toxic effect of AntQ in *E*. *coli* strains, wild type (MG1655) and a strain deleted of all genetic islands phages and insertion elements (MDS42). The strains were transformed with control and *antQ* expressing plasmids. IPTG (0.2 mM) to induce expression of *antQ* was added at dilution. OD600 values were measured at indicated times. (B) Survival assay. The cultures as above were treated with IPTG (0.2 mM) 60 minutes after dilution. Samples were taken prior to the addition of IPTG and 20 and 40 minutes after its addition. Survival rates were calculated using the CFU of the first time point as the 100% reference.
(PNG)

S13 Fig Protein profile changes in wild type cells expressing *antQ*. SDS-PAGE analysis of total protein extracted from wild type cells carrying control plasmid (P*tac*) or *antQ* expressing plasmids. The cultures were grown and induced with IPTG, as described in Materials and Methods. Arrow indicates the area (band) taken for mass-spec analysis.
(PNG)

S14 Fig Constitutive expression of *orf45* does not prevent *isrK* mediated growth inhibition of *Salmonella*. (A) Growth curves of *Salmonella* cells carrying plasmids expressing *orf45* and/or *isrK* as indicated. In the absence of *tetR*, *orf45* is expressed constitutively. *isrK* expression was induced by arabinose as described in the Materials and Methods. (B) Northern analysis of *orf45* RNA levels from the PL*tetO* promoter in the absence of *tetR*. The membrane was probed with fully labeled antisense of *orf45’*. The riboprobe detects the long RNA species extending from at the transcription start site of PL*tetO* to the plasmid encoded T1 terminator. Also visible is the short RNA species (STnc1160) that is generated by processing of the long transcript. STn1160 [8] is a truncated form *orf45* and thus denoted *orf45’*. The first two lanes also appear in S3 Fig. 5S RNA serves as a loading control.
(PNG)

S15 Fig RT-PCR to estimate expression levels of chromosomally and plasmid encoded *isrK*. Two samples per treatment and two reactions per sample were analyzed. (A) IsrK RNA levels increase at stationary phase and under low magnesium conditions. To monitor expression of chromosomally encoded-*isrK*, wild type and (ΔP*isrK*::*frt*) mutant strains were grown in LB medium to OD600 ~ 0.3, 0.6, 1.0 and for 8 hours or in low MgCl_2_ N-minimal medium to OD600 ~ 0.3 [6]. (B) Estimation of plasmid encoded *isrK* from PBAD promoter after induction in (ΔP*isrK*::*frt*) mutant strain. (C) Gifsy-1 prophage induction requires expression of the chromosomally encoded *isrK*. Wild type and (ΔP*isrK*::*frt*) mutant strains were grown in minimal medium to stationary phase. Thereafter, phage particles were collected and 15μ of the supernatant were plated on LT2 (lambda sensitive) as described in Materials and Methods. To confirm expression of *isrK* under these conditions, RNA samples were subjected to RT-PCR as described before.
(PNG)

S1 Table LacZ assays. (DOCX)

S2 Table Strains. (DOCX)

S3 Table Plasmids. (DOCX)

S4 Table DNA oligonucleotides. (DOCX)

S1 Text Additional references. (DOCX)

## References

[1] Wagner EG, Romby P (2015) Small RNAs in Bacteria and Archaea: Who They Are, What They Do, and How They Do It. Adv Genet 90:133–208. 10.1016/bs.adgen.2015.05.001 . 26296935

[2] Gottesman S, Storz G (2011) Bacterial small RNA regulators: versatile roles and rapidly evolving variations. Cold Spring Harb Perspect Biol 3(12). 10.1101/cshperspect.a003798 . 20980440 PMC3225950

[3] Vogel J, Luisi BF (2011) Hfq and its constellation of RNA. Nat Rev Microbiol 9(8):578–89. 10.1038/nrmicro2615 . 21760622 PMC4615618

[4] Vogel J, Bartels V, Tang TH, Churakov G, Slagter-Jäger JG, Hüttenhofer A, et al (2003) RNomics in *Escherichia coli* detects new sRNA species and indicates parallel transcriptional output in bacteria. Nucleic Acids Res 31(22):6435–43. . 14602901 10.1093/nar/gkg867 PMC275561

[5] Pichon C, Felden B (2005) Small RNA genes expressed from *Staphylococcus aureus* genomic and pathogenicity islands with specific expression among pathogenic strains. Proc Natl Acad Sci USA 102(40):14249–54. 10.1073/pnas.0503838102 . 16183745 PMC1242290

[6] Padalon-Brauch G, Hershberg R, Elgrably-Weiss M, Baruch K, Rosenshine I, Margalit H, et al (2008) Small RNAs encoded within genetic islands of *Salmonella typhimurium* show host-induced expression and role in virulence. Nucleic Acids Res 36(6):1913–27. 10.1093/nar/gkn050 . 18267966 PMC2330248

[7] Chao Y, Papenfort K, Reinhardt R, Sharma CM, Vogel J (2012) An atlas of Hfq-bound transcripts reveals 3' UTRs as a genomic reservoir of regulatory small RNAs. Embo j 31(20):4005–19. 10.1038/emboj.2012.229 . 22922465 PMC3474919

[8] Kröger C, Colgan A, Srikumar S, Händler K, Sivasankaran SK, Hammarlöf DL, et al (2013) An infection-relevant transcriptomic compendium for *Salmonella enterica* Serovar Typhimurium. Cell Host Microbe 14(6):683–95. 10.1016/j.chom.2013.11.010 . 24331466

[9] Bossi L, Fuentes JA, Mora G, Figueroa-Bossi N (2003) Prophage contribution to bacterial population dynamics. J Bacteriol 185(21):6467–71. 14563883 10.1128/JB.185.21.6467-6471.2003 PMC219396

[10] Figueroa-Bossi N, Uzzau S, Maloriol D, Bossi L (2001) Variable assortment of prophages provides a transferable repertoire of pathogenic determinants in *Salmonella*. Mol Microbiol 39(2):260–71. . 11136448 10.1046/j.1365-2958.2001.02234.x

[11] Miao EA, Miller SI (2000) A conserved amino acid sequence directing intracellular type III secretion by *Salmonella typhimurium*. Proc Natl Acad Sci USA 97(13):7539–44. . 10861017 10.1073/pnas.97.13.7539 PMC16581

[12] De Groote MA, Ochsner UA, Shiloh MU, Nathan C, McCord JM, Dinauer MC, et al (1997) Periplasmic superoxide dismutase protects *Salmonella* from products of phagocyte NADPH-oxidase and nitric oxide synthase. Proc Natl Acad Sci USA 94(25):13997–4001. . 9391141 10.1073/pnas.94.25.13997 PMC28421

[13] Farrant JL, Sansone A, Canvin JR, Pallen MJ, Langford PR, Wallis TS, et al (1997) Bacterial copper- and zinc-cofactored superoxide dismutase contributes to the pathogenesis of systemic salmonellosis. Mol Microbiol 25(4):785–96. . 9379906 10.1046/j.1365-2958.1997.5151877.x

[14] Stanley TL, Ellermeier CD, Slauch JM (2000) Tissue-specific gene expression identifies a gene in the lysogenic phage Gifsy-1 that affects *Salmonella enterica* serovar Typhimurium survival in Peyer's patches. J Bacteriol 182(16):4406–13. . 10913072 10.1128/jb.182.16.4406-4413.2000 PMC94610

[15] Figueroa-Bossi N, Bossi L (1999) Inducible prophages contribute to *Salmonella* virulence in mice. Mol Microbiol 33(1):167–76. . 10411733 10.1046/j.1365-2958.1999.01461.x

[16] Hébrard M, Kröger C, Srikumar S, Colgan A, Händler K, Hinton JC (2012) sRNAs and the virulence of *Salmonella enterica* serovar Typhimurium. RNA Biol 9(4):437–45. 10.4161/rna.20480 . 22546935 PMC3384567

[17] Romby P, Vandenesch F, Wagner EG (2006) The role of RNAs in the regulation of virulence-gene expression. Curr Opin Microbiol 9(2):229–36. 10.1016/j.mib.2006.02.005 . 16529986

[18] Pfeiffer V, Sittka A, Tomer R, Tedin K, Brinkmann V, Vogel J (2007) A small non-coding RNA of the invasion gene island (SPI-1) represses outer membrane protein synthesis from the *Salmonella* core genome. Mol Microbiol 66(5):1174–91. 10.1111/j.1365-2958.2007.05991.x . 17971080

[19] Massé E, Vanderpool CK, Gottesman S (2005) Effect of RyhB small RNA on global iron use in *Escherichia coli*. J Bacteriol 187(20):6962–71. 10.1128/jb.187.20.6962–6971.2005 . 16199566 PMC1251601

[20] Deighan P, Hochschild A (2007) The bacteriophage lambdaQ anti-terminator protein regulates late gene expression as a stable component of the transcription elongation complex. Mol Microbiol 63(3):911–20. 10.1111/j.1365-2958.2006.05563.x . 17302807

[21] Shankar S, Hatoum A, Roberts JW (2007) A transcription antiterminator constructs a NusA-dependent shield to the emerging transcript. Mol Cell 27(6):914–27. 10.1016/j.molcel.2007.07.025 . 17889665 PMC2075354

[22] Yarnell WS, Roberts JW (1999) Mechanism of intrinsic transcription termination and antitermination. Science 284(5414):611–5. . 10213678 10.1126/science.284.5414.611

[23] Roberts JW, Yarnell W, Bartlett E, Guo J, Marr M, Ko DC, et al (1998) Antitermination by bacteriophage lambda Q protein. Cold Spring Harb Symp Quant Biol 63:319–25. . 10384296 10.1101/sqb.1998.63.319

[24] Roberts JW, Shankar S, Filter JJ (2008) RNA polymerase elongation factors. Annu Rev Microbiol 62:211–33. 10.1146/annurev.micro.61.080706.093422 . 18729732 PMC2819089

[25] Friedman DI, Court DL (1995) Transcription antitermination: the lambda paradigm updated. Mol Microbiol 18(2):191–200. . 8709839 10.1111/j.1365-2958.1995.mmi_18020191.x

[26] Lemire S, Figueroa-Bossi N, Bossi L (2011) Bacteriophage crosstalk: coordination of prophage induction by trans-acting antirepressors. PLoS Genet 7(6):e1002149 10.1371/journal.pgen.1002149 . 21731505 PMC3121763

[27] Zeghouf M, Li J, Butland G, Borkowska A, Canadien V, Richards D, et al (2004) Sequential Peptide Affinity (SPA) system for the identification of mammalian and bacterial protein complexes. J Proteome Res 3(3):463–8. . 15253427 10.1021/pr034084x

[28] Pósfai G, Plunkett G 3rd, Fehér T, Frisch D, Keil GM, Umenhoffer K, et al (2006) Emergent properties of reduced-genome *Escherichia coli*. Science 312(5776):1044–6. 10.1126/science.1126439 . 16645050

[29] Peters JM, Mooney RA, Kuan PF, Rowland JL, Keles S, Landick R (2009) Rho directs widespread termination of intragenic and stable RNA transcription. Proc Natl Acad Sci USA 106(36):15406–11. 10.1073/pnas.0903846106 . 19706412 PMC2741264

[30] Dutta D, Shatalin K, Epshtein V, Gottesman ME, Nudler E (2011) Linking RNA polymerase backtracking to genome instability in *E*. *coli*. Cell 146(4):533–43. 10.1016/j.cell.2011.07.034 . 21854980 PMC3160732

[31] Pomerantz RT, O'Donnell M (2010) What happens when replication and transcription complexes collide? Cell Cycle 9(13):2537–43. 10.4161/cc.9.13.12122 . 20581460 PMC3918965

[32] Ciampi MS (2006) Rho-dependent terminators and transcription termination. Microbiology 152(Pt 9):2515–28. 10.1099/mic.0.28982–0 . 16946247

[33] Helmrich A, Ballarino M, Nudler E, Tora L (2013) Transcription-replication encounters, consequences and genomic instability. Nat Struct Mol Biol 20(4):412–8. 10.1038/nsmb.2543 . 23552296

[34] Merrikh H, Zhang Y, Grossman AD, Wang JD (2012) Replication-transcription conflicts in bacteria. Nat Rev Microbiol 10(7):449–58. 10.1038/nrmicro2800 . 22669220 PMC3467967

[35] Aguilera A, García-Muse T (2012) R loops: from transcription byproducts to threats to genome stability. Mol Cell 46(2):115–24. 10.1016/j.molcel.2012.04.009 . 22541554

[36] Shechter N, Zaltzman L, Weiner A, Brumfeld V, Shimoni E, Fridmann-Sirkis Y, et al (2013) Stress-induced condensation of bacterial genomes results in re-pairing of sister chromosomes: implications for double strand DNA break repair. J Biol Chem 288(35):25659–67. 10.1074/jbc.M113.473025 . 23884460 PMC3757227

[37] Epshtein V, Kamarthapu V, McGary K, Svetlov V, Ueberheide B, Proshkin S, et al (2014) UvrD facilitates DNA repair by pulling RNA polymerase backwards. Nature 505(7483):372–7. 10.1038/nature12928 . 24402227 PMC4471481

[38] Boubakri H, de Septenville AL, Viguera E, Michel B (2010) The helicases DinG, Rep and UvrD cooperate to promote replication across transcription units in *vivo*. Embo j 29(1):145–57. 10.1038/emboj.2009.308 . 19851282 PMC2770101

[39] Gwynn EJ, Smith AJ, Guy CP, Savery NJ, McGlynn P, Dillingham MS (2013) The conserved C-terminus of the PcrA/UvrD helicase interacts directly with RNA polymerase. PLoS One 8(10):e78141 10.1371/journal.pone.0078141 . 24147116 PMC3797733

[40] Taniguchi Y, Choi PJ, Li GW, Chen H, Babu M, Hearn J, et al (2010) Quantifying *E*. *coli* proteome and transcriptome with single-molecule sensitivity in single cells. Science 329(5991):533–8. 10.1126/science.1188308 . 20671182 PMC2922915

[41] Spanjaard RA, van Duin J (1989) Translational reinitiation in the presence and absence of a Shine and Dalgarno sequence. Nucleic Acids Res 17(14):5501–7. . 2668889 10.1093/nar/17.14.5501 PMC318173

[42] Oppenheim DS, Yanofsky C (1980) Translational coupling during expression of the tryptophan operon of *Escherichia coli*. Genetics 95(4):785–95. . 6162715 10.1093/genetics/95.4.785 PMC1214269

[43] Sonnleitner E, Gonzalez N, Sorger-Domenigg T, Heeb S, Richter AS, Backofen R, et al (2011) The small RNA PhrS stimulates synthesis of the *Pseudomonas aeruginosa* quinolone signal. Mol Microbiol 80(4):868–85. 10.1111/j.1365-2958.2011.07620.x . 21375594

[44] Barquist L, Langridge GC, Turner DJ, Phan MD, Turner AK, Bateman A, et al (2013) A comparison of dense transposon insertion libraries in the *Salmonella* serovars Typhi and Typhimurium. Nucleic Acids Res 41(8):4549–64. 10.1093/nar/gkt148 . 23470992 PMC3632133

[45] Ghisotti D, Chiaramonte R, Forti F, Zangrossi S, Sironi G, Dehò G (1992) Genetic analysis of the immunity region of phage-plasmid P4. Mol Microbiol 6(22):3405–13. . 1484492 10.1111/j.1365-2958.1992.tb02208.x

[46] Sabbattini P, Forti F, Ghisotti D, Dehò G (1995) Control of transcription termination by an RNA factor in bacteriophage P4 immunity: identification of the target sites. J Bacteriol 177(6):1425–34. . 7883698 10.1128/jb.177.6.1425-1434.1995 PMC176756

[47] Briani F, Ghisotti D, Dehò G (2000) Antisense RNA-dependent transcription termination sites that modulate lysogenic development of satellite phage P4. Mol Microbiol 36(5):1124–34. . 10844696 10.1046/j.1365-2958.2000.01927.x

[48] Guo MS, Updegrove TB, Gogol EB, Shabalina SA, Gross CA, Storz G (2014) MicL, a new sigmaE-dependent sRNA, combats envelope stress by repressing synthesis of Lpp, the major outer membrane lipoprotein. Genes Dev 28(14):1620–34. 10.1101/gad.243485.114 . 25030700 PMC4102768

[49] Loh E, Dussurget O, Gripenland J, Vaitkevicius K, Tiensuu T, Mandin P, et al (2009) A trans-acting riboswitch controls expression of the virulence regulator PrfA in *Listeria monocytogenes*. Cell 139(4):770–9. 10.1016/j.cell.2009.08.046 . 19914169

[50] Sayed N, Jousselin A, Felden B (2012) A cis-antisense RNA acts in trans in *Staphylococcus aureus* to control translation of a human cytolytic peptide. Nat Struct Mol Biol 19(1):105–12. 10.1038/nsmb.2193 . 22198463

[51] Jäger D, Pernitzsch SR, Richter AS, Backofen R, Sharma CM, Schmitz RA (2012) An archaeal sRNA targeting cis- and trans-encoded mRNAs via two distinct domains. Nucleic Acids Res 40(21):10964–79. 10.1093/nar/gks847 . 22965121 PMC3510493

[52] Yu D, Ellis HM, Lee EC, Jenkins NA, Copeland NG, Court DL (2000) An efficient recombination system for chromosome engineering in *Escherichia coli*. Proc Natl Acad Sci USA 97(11):5978–83. 10.1073/pnas.100127597 . 10811905 PMC18544

[53] Datsenko KA, Wanner BL (2000) One-step inactivation of chromosomal genes in *Escherichia coli* K-12 using PCR products. Proc Natl Acad Sci USA 97(12):6640–5. 10.1073/pnas.120163297 . 10829079 PMC18686

[54] Maloy SR (1996) Genetic Analysis of Pathogenic Bacteria: a Laboratory Manual. SV J., editor. New York: Cold Spring Harbor Laboratory Press.

[55] Ho C, Kulaeva OI, Levine AS, Woodgate R (1993) A rapid method for cloning mutagenic DNA repair genes: isolation of umu-complementing genes from multidrug resistance plasmids R391, R446b, and R471a. J Bacteriol 175(17):5411–9. . 8366028 10.1128/jb.175.17.5411-5419.1993 PMC206596

[56] Nechooshtan G, Elgrably-Weiss M, Sheaffer A, Westhof E, Altuvia S (2009) A pH-responsive riboregulator. Genes Dev 23(22):2650–62. 10.1101/gad.552209 . 19933154 PMC2779765

[57] Lutz R, Bujard H (1997) Independent and tight regulation of transcriptional units in *Escherichia coli* via the LacR/O, the TetR/O and AraC/I1-I2 regulatory elements. Nucleic Acids Res 25(6):1203–10. . 9092630 10.1093/nar/25.6.1203 PMC146584

[58] Miller JH (1972) Experiments in Molecular Genetics Cold Spring Harbor. New York: Cold Spring Harbor Laboratory.

[59] Frye JG, Porwollik S, Blackmer F, Cheng P, McClelland M (2005) Host gene expression changes and DNA amplification during temperate phage induction. J Bacteriol 187(4):1485–92. 10.1128/jb.187.4.1485–1492.2005 . 15687213 PMC545606

[60] Hemm MR, Paul BJ, Schneider TD, Storz G, Rudd KE (2008) Small membrane proteins found by comparative genomics and ribosome binding site models. Mol Microbiol 70(6):1487–501. 10.1111/j.1365-2958.2008.06495.x . 19121005 PMC2614699

[61] Govindarajan S, Elisha Y, Nevo-Dinur K, Amster-Choder O (2013) The general phosphotransferase system proteins localize to sites of strong negative curvature in bacterial cells. MBio 4(5):e00443–13. 10.1128/mBio.00443-13 24129255 PMC3812706

