# Correction: Gifsy-1 Prophage IsrK with Dual Function as Small and Messenger RNA Modulates Vital Bacterial Machineries

**DOI:** 10.1371/journal.pgen.1006725

**Published:** 2017-04-14

**Authors:** Tal Hershko-Shalev, Ahuva Odenheimer-Bergman, Maya Elgrably-Weiss, Tamar Ben-Zvi, Sutharsan Govindarajan, Hemda Seri, Kai Papenfort, Jörg Vogel, Shoshy Altuvia

Data were missing from S3 Table. Please view the correct S3 Table here.

## Supporting information

S3 Table Plasmids. (DOCX)
