# Supplementary figures and images for: Gifsy-1 Prophage IsrK with Dual Function as Small and Messenger RNA Modulates Vital Bacterial Machineries
Source: PLoS Genet. 2016 Apr 8;12(4):e1005975. doi: 10.1371/journal.pgen.1005975 (PMC4825925; doi:10.1371/journal.pgen.1005975)

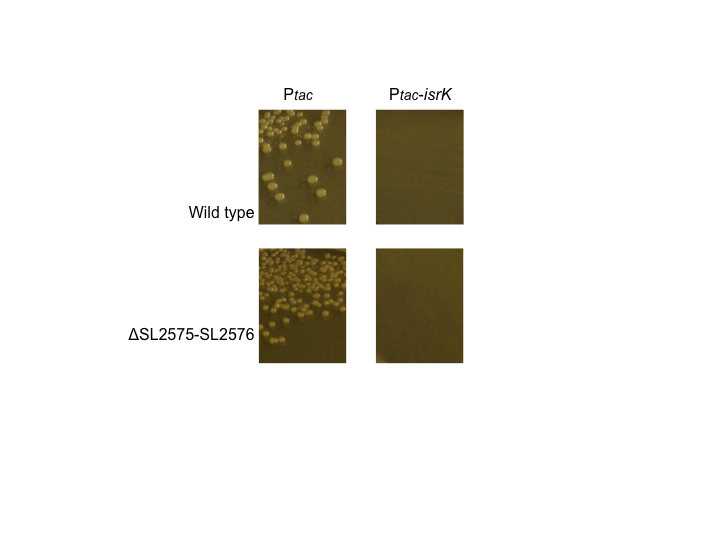

Supplement: S1 Fig — Salmonella wild type and cells deleted of SL2575 and SL2576 genes encoding proteins of phage lysis and phage lysozyme superfamily, respectively, were transformed with Ptac plasmid and Ptac-isrK, expressing IsrK constitutively. (PNG) [file pgen.1005975.s001.png]

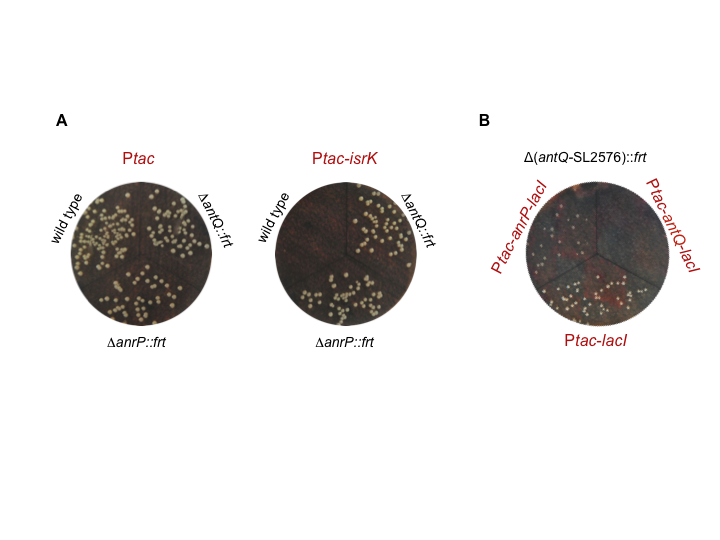

Supplement: S2 Fig — Salmonella, wild type, ΔantQ::frt and ΔanrP::frt were transformed with Ptac plasmid and Ptac-isrK expressing IsrK constitutively (A). (B) Salmonella deleted of the entire locus between antQ and the Gifsy-1 lysis genes SL2575-SL2576 (including) was transformed with plasmids expressing anrP or antQ from Ptac promoter under the control of LacI repressor (pKK177-3-lacI). The transformations were plated on LB plates supplemented with IPTG (0.05 mM) and antibiotics. Plasmids are denoted in red. (PNG) [file pgen.1005975.s002.png]

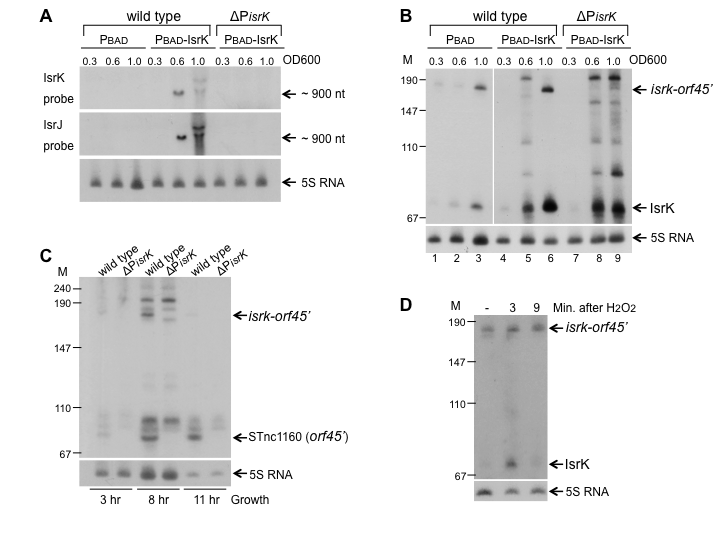

Supplement: S3 Fig — Northern blot of RNA extracted from wild type and cells deleted of the isrK promoter in the chromosome (ΔPisrK) as well as cells carrying a control plasmid (PBAD) or an isrK expressing plasmid (PBAD-isrK) as indicated. Cultures carrying plasmids were induced with arabinose at dilution. (A) 1% agarose formaldehyde gel detecting the full-length transcript isrK-orf45-anrP-isrJ. The membrane was probed with 5’-end labeled IsrK and IsrJ specific primers. (B) 6% urea-PAGE detecting short IsrK (77nt) both chromosomally (lanes 1–3) and plasmid encoded (lanes 4–9). It is interesting to note that the northern also detects a stable transcript (isrK-orf45’) that was generated by processing of the long polycistronic transcript, (lanes 1–3 and 6). The membrane was probed with end-labeled isrK specific primer. The left panel (lanes 1–3) was exposed longer (3x) than the right panel. (C) 6% urea-PAGE carrying RNA extracted from cultures of wild type and ΔPisrK grown for 3 (OD600 of 1.0), 8 and 11 hours as indicated. The membrane was probed with fully labeled antisense of orf45’. The riboprobe detects two RNA species that are generated by processing of the read-through transcript; a ~180 nt long RNA (isrk-orf45’, also detected by isrK primer in (A) and a small species of ~ 80 nt that is generated by further processing of isrk-orf45’. The truncated RNA species (orf45’) was also detected in Salmonella’s transcriptome (see Discussion STnc1160 [8]). (D) Oxidative stress induces expression of isrK. Both short isrK and isrK-orf45’ that is generated by processing of the long polycistronic transcript are detected in response to exposure to hydrogen peroxide (see Materials and Methods for details). The membrane was probed with end-labeled isrK specific primer. 5S RNA serves as a loading control. (PNG) [file pgen.1005975.s003.png]

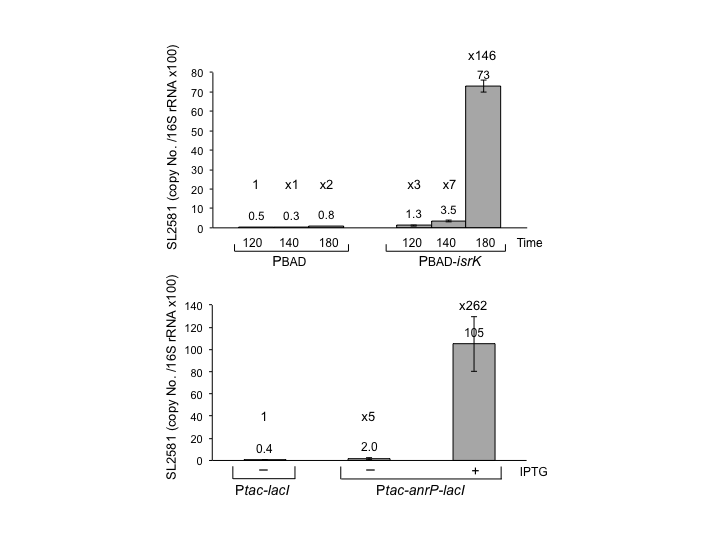

Supplement: S4 Fig — Real-Time PCR of SL2581 mRNA detected in the presence of high levels of IsrK (PBAD-isrK) (A) or AnrP (Ptac-anrP-lacI) (B). SL2581 is the second gene in antQ operon [8] that includes SL2582, SL2581, SL2580 and SL2579 (here denoted antQ). Salmonella carrying control, IsrK, and AnrP expressing plasmids were exposed to arabinose and IPTG to activate PBAD and Ptac promoters, respectively (see also Materials and Methods). Two samples per treatment and two reactions per sample were analyzed. (PNG) [file pgen.1005975.s004.png]

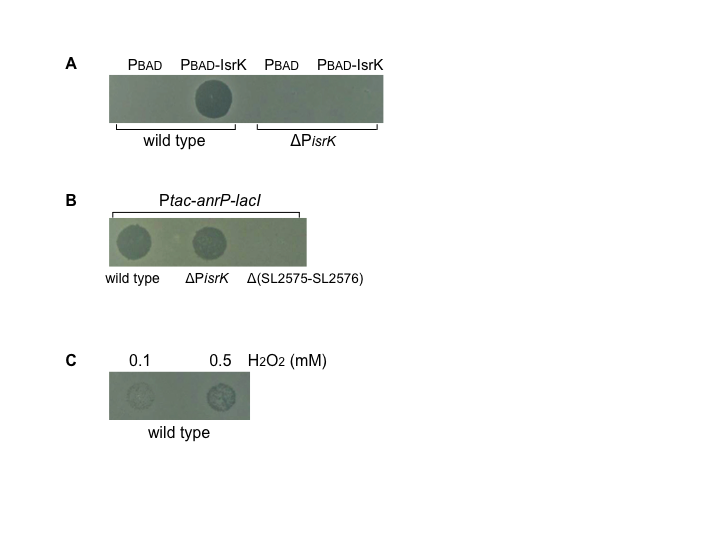

Supplement: S5 Fig — (A) Gifsy-1 phage induction by IsrK requires an intact isrK locus. Cultures of S. typhimurium wild type and isrK promoter deletion mutant (ΔPisrK::frt) carrying PBAD and PBAD-isrK were grown with arabinose to induce isrK expression for two hours. Thereafter, their phages were released by chloroform and plated on LT2 (lambda sensitive) as described in Materials and Methods. (B) Gifsy-1 phage induction by AnrP is independent of isrK locus. Wild type, (ΔPisrK::frt), and Gifsy-1 lysis proteins deletion mutant (ΔSL2575-SL2576::frt) carrying Ptac-anrP-lacI were grown with IPTG to induce expression of anrP and their phages were collected and plated on LT2 (lambda sensitive) as described in Materials and Methods. (C) Oxidative stress dependent phage induction. H2O2 (0.1 and 0.5 mM) was added at OD600 ~ 0.3 and phages were plated as above. (PNG) [file pgen.1005975.s005.png]

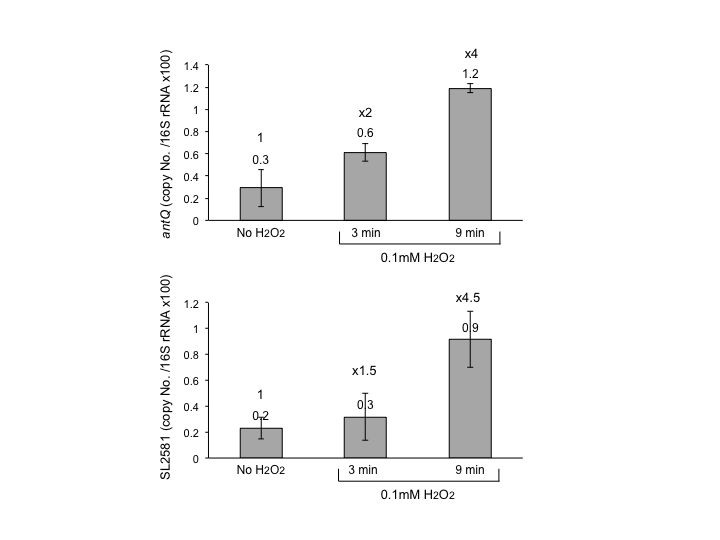

Supplement: S6 Fig — Real-Time PCR of antQ and SL2581 mRNAs expressed upon phage induction by hydrogen peroxide as indicated in the Fig. Samples were taken and assayed as described in Materials and Methods. SL2581 is the second gene in antQ operon [8] that includes SL2582, SL2581, SL2580 and SL2579 (here denoted antQ). Two samples per treatment and two reactions per sample were analyzed. (PNG) [file pgen.1005975.s006.png]

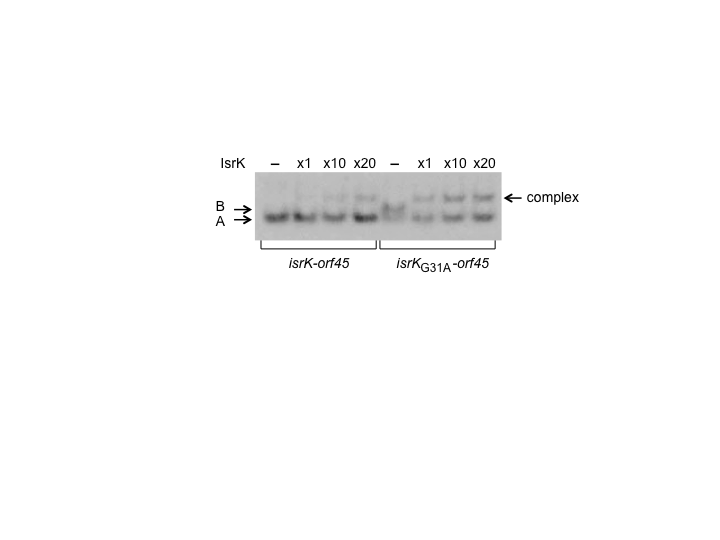

Supplement: S9 Fig — RNAs (0.2 pmol), wild type isrK-orf45 and mutant isrKG31A-orf45 were incubated for 15 minutes at 37°C in the presence of increasing amounts of IsrK, as indicated. The samples were separated on non-denaturing polyacrylamide gels. Arrows indicate the two conformations observed. The target-RNAs were detected using an orf45 specific labeled primer (1948). Analysis of the RNA samples on denaturing gels exhibits one form (see Fig 6C). (PNG) [file pgen.1005975.s009.png]

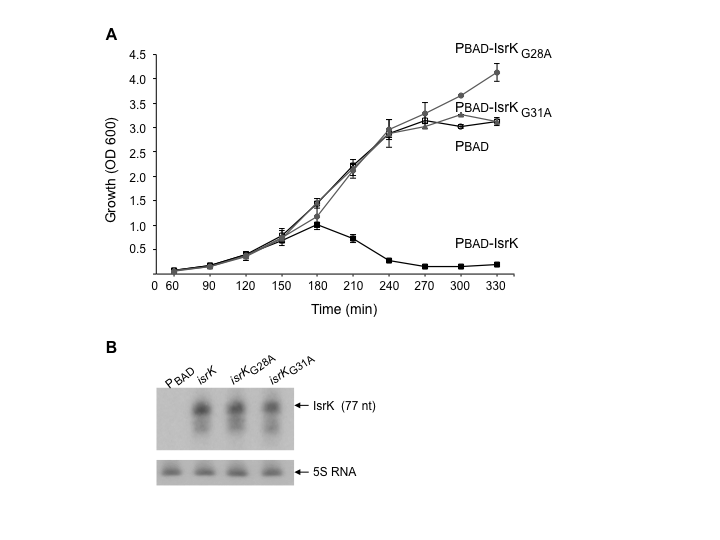

Supplement: S10 Fig — (A) Growth curves of wild type cells carrying control (PBAD) or isrK expressing plasmids; wild type, isrKG28A and isrKG31. (B) Northern blot comparing RNA levels of wild type and isrK mutants. RNA was extracted from cells deleted of the isrK locus (ΔisrK to isrJ) carrying plasmids as in A. Cells were induced with arabinose (0.2%) at the time of dilution and RNA was extracted at OD600 1.0 (PNG) [file pgen.1005975.s010.png]

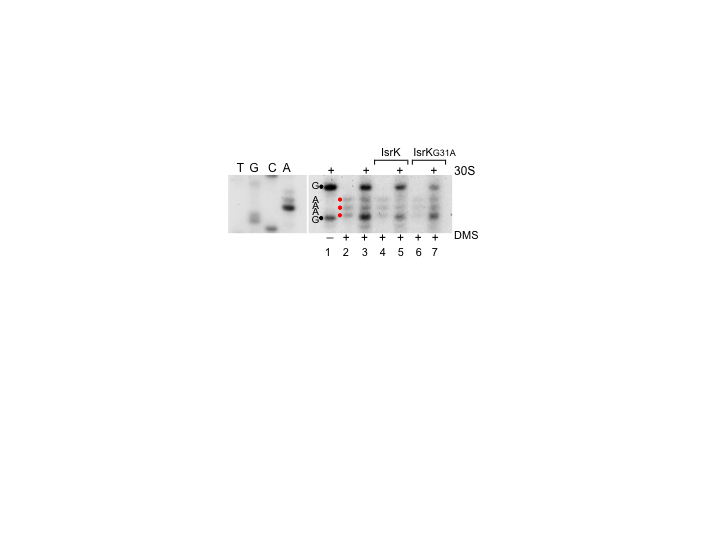

Supplement: S11 Fig — In vitro synthesized RNA templates were incubated with and without 30S ribosomes, IsrK RNA or IsrKG31A prior to the addition of DMS. Thereafter, the samples were treated with phenol as described in Materials and Methods. The modified sites were detected by primer extension. In this experiment, we used increased concentrations of 30S ribosomal subunits (2.4 pmol). Under these conditions, primer extension termination sites can be detected at the marked G residues in the absence of DMS (lane 1). The authentic DMS modification sites at AAA residues are marked by red dots. (PNG) [file pgen.1005975.s011.png]

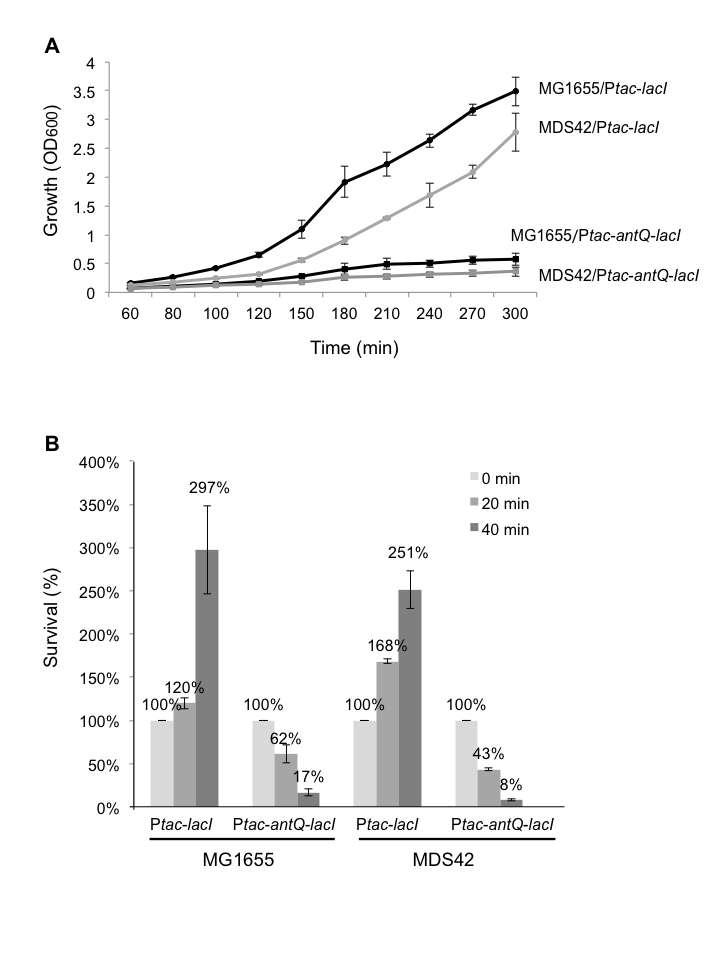

Supplement: S12 Fig — Growth curves (A) showing the toxic effect of AntQ in E. coli strains, wild type (MG1655) and a strain deleted of all genetic islands phages and insertion elements (MDS42). The strains were transformed with control and antQ expressing plasmids. IPTG (0.2 mM) to induce expression of antQ was added at dilution. OD600 values were measured at indicated times. (B) Survival assay. The cultures as above were treated with IPTG (0.2 mM) 60 minutes after dilution. Samples were taken prior to the addition of IPTG and 20 and 40 minutes after its addition. Survival rates were calculated using the CFU of the first time point as the 100% reference. (PNG) [file pgen.1005975.s012.png]

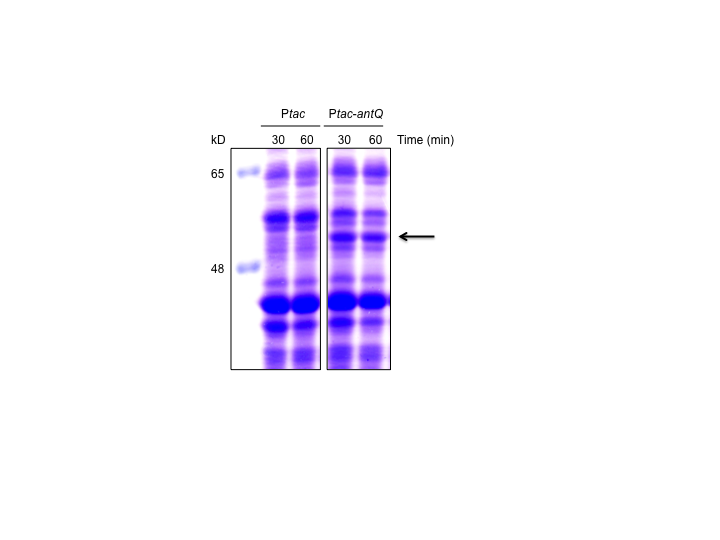

Supplement: S13 Fig — SDS-PAGE analysis of total protein extracted from wild type cells carrying control plasmid (Ptac) or antQ expressing plasmids. The cultures were grown and induced with IPTG, as described in Materials and Methods. Arrow indicates the area (band) taken for mass-spec analysis. (PNG) [file pgen.1005975.s013.png]

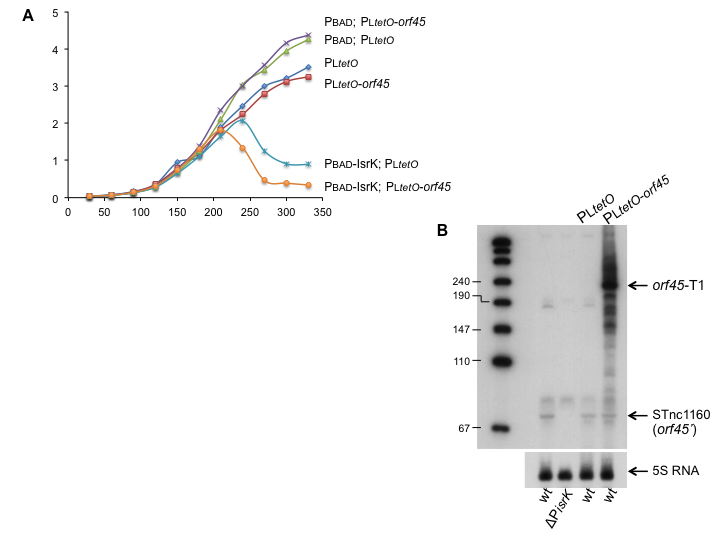

Supplement: S14 Fig — (A) Growth curves of Salmonella cells carrying plasmids expressing orf45 and/or isrK as indicated. In the absence of tetR, orf45 is expressed constitutively. isrK expression was induced by arabinose as described in the Materials and Methods. (B) Northern analysis of orf45 RNA levels from the PLtetO promoter in the absence of tetR. The membrane was probed with fully labeled antisense of orf45’. The riboprobe detects the long RNA species extending from at the transcription start site of PLtetO to the plasmid encoded T1 terminator. Also visible is the short RNA species (STnc1160) that is generated by processing of the long transcript. STn1160 [8] is a truncated form orf45 and thus denoted orf45’. The first two lanes also appear in S3 Fig. 5S RNA serves as a loading control. (PNG) [file pgen.1005975.s014.png]

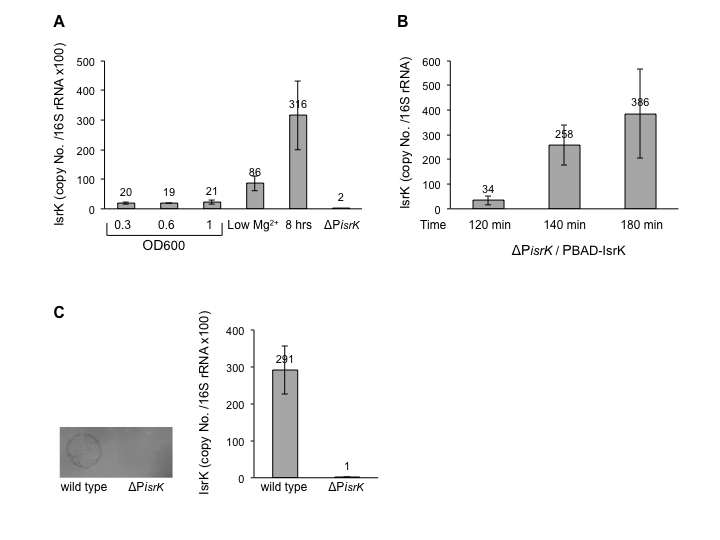

Supplement: S15 Fig — Two samples per treatment and two reactions per sample were analyzed. (A) IsrK RNA levels increase at stationary phase and under low magnesium conditions. To monitor expression of chromosomally encoded-isrK, wild type and (ΔPisrK::frt) mutant strains were grown in LB medium to OD600 ~ 0.3, 0.6, 1.0 and for 8 hours or in low MgCl2 N-minimal medium to OD600 ~ 0.3 [6]. (B) Estimation of plasmid encoded isrK from PBAD promoter after induction in (ΔPisrK::frt) mutant strain. (C) Gifsy-1 prophage induction requires expression of the chromosomally encoded isrK. Wild type and (ΔPisrK::frt) mutant strains were grown in minimal medium to stationary phase. Thereafter, phage particles were collected and 15μ of the supernatant were plated on LT2 (lambda sensitive) as described in Materials and Methods. To confirm expression of isrK under these conditions, RNA samples were subjected to RT-PCR as described before. (PNG) [file pgen.1005975.s015.png]
