# Supplementary material for: Gifsy-1 Prophage IsrK with Dual Function as Small and Messenger RNA Modulates Vital Bacterial Machineries
Source: PLoS Genet. 2016 Apr 8;12(4):e1005975. doi: 10.1371/journal.pgen.1005975 (PMC4825925; doi:10.1371/journal.pgen.1005975)
Supplement: S7 Fig — Representative genera from the original analysis (blast) are shown. Initiation and stop codons of orf45 and anrP are marked in red. Complementary nucleotides between isrK and orf45 are marked in green. The black arrows under the green sequences denote the position and the orientation of basepairing. (PDF) [file pgen.1005975.s007.pdf]

Nucleotide conservation of IsrK sRNA (+1 +77)

```
S. Typhimurium   ACGCCAGCAAAATCTGGCGTCGGGATT GGCGTCCCGGAATTTC AC  CGCGACA  GAGACACGCC GCGAGCGTGTTTTTT
S. Newport       ACGCCAGCAAAATCTGGCGTCGGGATT GGCGTCCCGGAATTTC AC  CGCGACA  GAGACACGCC GCGAGCGTGTTTTTT
E. coli ED1a     ACGCCAGCAAAATCTGGCGTCGGGATTAGGAA CCCCGGATAGAAAC  CGCGACA  GAGACACGCC GCGAGCGTGTTTTTT
E. coli RM9387       CAGCAAAATCTGGCGTCGGGATT GGAA CCCCGGATGTTTACGGAGCGATAT GAGACGCGCCCGCG    TCTTTTTT
S. dysenteriae   ACGCCAGCAAAATCTGGCGTCGGGATT  GAGACCCCGGATGTTTACGGAGCGATAT GAGACGCGCCCGCG    TCTTTTTT
Conservation         ***********************  *   ********    **   **** *  ***** **** ***      ******
```

Nucleotide conservation (+78 to +106)

```
S. Typhimurium   ATTGTCGTTTGTATACGCGCATT  TGAATT
S. Newport       ATTGTCGTTTGTATACGCGCATT  TGAATT
E. coli ED1a     ATTGTCGTATGCACACGCACATC  TGAATT
E. coli RM9387   CATATCGTTTGCACAGTCACATTCGCGATTT
S. dysenteriae   AT ATCGTTTGCACAGTCACATTCGCGATTT
Conservation         **** ** * *    ***    ** **
```

Nucleotide conservation of *orf45* (107-138)

```
S. Typhimurium   ATGGTGGGGCGTATGGGGGAGCCGAAAGGCTCGCCGG  TCGGTGATCC  GGTTAC GCC AACCCTGTACGTCT
S. Newport       ATGGTGGGGCGTATGGGGGAGCCGAAAGGCTCGCCGG  TCGGTGATCC  GGTTAC GCC AACCCTGTACGTCT
E. coli ED1a     ATGGTGGGGCGTATAGGGGAGCTGAAAAGCTCGCCGG  TTGGTTTCCC  GGTAGTTCCT AACCCTGTACGTCT
E. coli RM9387   ATGGCGGGCTGTGTGGGGGAGCCGAAAGGCTCGCCGG  TTTCCGTACCC GGTAGTT CC  AACCCCGCACAGTT
S. dysenteriae   ATGGTGGGCTGTGTGGGGGAGCCGAAAGGCTCGCCGG  TTTCCGTACCC GGTAGTCTC  AACCCTGCACAGCT
Conservation     **** ***  ** ************************  *       **   **        ******* **   *

S. Typhimurium   CACCACCCAATCCGATT GGCGTCGGCGGTGGTGATAAACAAAACAATCACCGGAGGGCGTCA TTATGA 138
S. Newport       CACCACCCAATCCGATT GGCGTCGGCGGTGGTGATAAACAAAACAATCACCGGAGGGCGTCA TTATGA 138
E. coli ED1a     CGCCACCCGA  TGATTAGGAACCTGACGGTGGTGATAGTTTAGAAACCACTCGAGGGCGTCA TTATGA 138
E. coli RM9387   CGCCACCATGA TGATT GGAACCTGAAGGTGGCGATAATTTTCAAATGTACGGAGTTA TCG TTATGA 137
S. dysenteriae   CGCCACCAGAT  TATT GAGACCAGAAGGTGGCGATAATGACCAAATGTACGGAGTTA TCG TTATGC 136
Conservation     * *****       *** *    * *  *  *     *       **      ***    **  *****
```

Nucleotide conservation (AnrP)

```
S.Typhimurium      ATGACCACTCAAATCTCTGTCGAAACTCTTTCCCCGATTACCCATAACCAAATTCCCGTTATTACTACCGAACTTTTGGC
S. Newport         ATGACCACTCAAATCTCTGTCGAAACTCTTTCCCCGATTACCCATAACCAAATTCCCGTTATTACTACCGAACTTTTGGC
E. coli ED1a       ATGACAACTCAAGTTTCTGTTGAAACACTCTCCACGATTACTTACAAGCAGATCCCCGTTATCACTACCGAACTTTTGGC
E. coli RM9387     ATGACCACTCAAATTTCTGTCGACACTCTTCCTGCAATCACCCACAACCAGATCCCCGTTATTACAACTGAACTTCTGGC
S. dysenteriae     ATGCTCACTCAAATTTCTGTCGACACTCTTCCTGCAATCACCCACAACCAGATCCGCGTTATTACAACTGAACTTCTGGC
Conservation       ***   ******** ***** ** ** *** *  * ** **   * ** ** ** * ****** ** ** ****** ****

S.Typhimurium      GCACTTATACGGCACAAAAATCAAAAACAT  TTCT   GA TAACTTTCTGAACAACACCACG CGATTCGTTGTAGGAA
S. Newport         GCACTTATACGGCACAAAAATCAAAAACAT  TTCT   GA TAACTTTCTGAACAACACCACG CGATTCGTTGTAGGAA
E. coli ED1a       GCACCTTTACGGCACAGAAGCTATTCGTA   TTCGCCAGAATCACCA C GAA AACAA AGGTCGTTTCATTG AGGAA
E. coli RM9387     GCAGTTATACGGAACAAAAATCAAAAACAT  TTCT   GA TAACTTTCTGAACAACACGACG CGATTCGTGGTAGGAA
S. dysenteriae     GCAGTTATACGGAACAAAAATCAAAAACAT  TTCT   GA TAATTTTCTGAACAACACGATG CGATTCGTGGTAGGAA
Conservation       ***  * ***** *** **   *     **  ***    ** * *    * *** ****  * * ** *** * * *****

S.Typhimurium      AG CATTTTTTTAAAATTGAAAA  AAACGAATTACGCGAGTTCAAGAACAGACCCGA     AACAA TCGG GTTAGTT
S. Newport         AG CATTTTTTTAAAATTGAAAA  AAACGAATTACGCGAGTTCAAGAACAGACCCGA     AACAA TCGG GTTAGTT
E. coli ED1a       AAACACTTCTTCAAACTTGAAGGTGAAACT   TTACGTGAGTTCAAGCACAGAGTAGCTTTTAACTACTCTGTGAAAATT
E. coli RM9387     AG CATTACTTTAAAATTGAAAA  AAACGAATTACGCGAGTTTAAGAACAGACCCGA     AACAA TCGG GTTAGTT
S. dysenteriae     AG CATTACTTTAAAATTGAAAA  AAACGAATTACGCGAGTTTAAGAACAGACCCGA     AACAA TCGG GTTAGTT
Conservation       *  ** *  **  *** *****    **** ******* ***** *** *****         *** * ** * *  * **

S.Typhimurium      GGTAAAAATGCCCGTTCCCTAATCCTCTGGACAGAACGCGGCGCTGCCCGCCACGCCAAGATGCTCGAAACAGATCAGG
S. Newport         GGTAAAAATGCCCGTTCCCTAATCCTCTGGACAGAACGCGGCGCTGCCCGCCACGCCAAGATGCTCGAAACAGATCAGG
E. coli ED1a       GCCCGTAACGTTCGCTCCCTCATCCTATGGACAGAACGCGGCGCAGCCCGTCATGCAAAAATGCTCGAAACCGATCGGG
E. coli RM9387     GGTAAAAATGCCCGCTCCCTTATCCTCTGGACAGAACGTGGCGCTGCCCGCCATGCAAAAATGCTCGAAACCGATCAGG
S. dysenteriae     GGTAAAAATGCCCGCTCCCTCATCCTCTGGACAGAACGCGGAGCCGCACGCCACGCCAAAATGCTCGAAACCGATCAGG
Conservation       *     ** *   ** ***** ***** *********** ** ** ** ** ** ** ** *********** *******

S.Typhimurium      CTTGGGAGGTGTTCGAAAAACTGGAGGATTGCTATTTCAGTCAGACACTACCATCGCCAACACGCCAGGTTCAGCCTGC
S. Newport         CTTGGGAGGTGTTCGAAAAACTGGAGGATTGCTATTTCAGTCAGACACTACCATCGCCAACACGCCAGGTTCAGCCTGC
E. coli ED1a       CGTGGGAAGTGTTCGAAAAACTGGAAGACTGCTATTTCAGCCAG-----------------------------------
E. coli RM9387     CGTGGGAAGTGTTCGAAAAACTGGAAGACTGTTATTTCAGCCATAAACAACCACCAGCAACAC----------------
S. dysenteriae     CGTGGGACGTGTTCGAAAAACTGGAAGACTGCTATTTCAGCCAGAC---------------------------------
Conservation       * ***** ***************** ** ** ******** ** *
```

Nucleotide conservation (AnrP)- Continue

```
S.Typhimurium     CGTCGACATGCTTAACATCGACCTTCTGATTAAGATCCGCGATGGTAACGTCAAAGACATTCGGCAGGTTGGT
S. Newport        CGTCGACATGCTTAACATCGACCTTCTGATTAAGATCCGCGATGGTAACGTCAAAGACATTCGGCAGGTTGGT
E. coli ED1a      -------------------------------------------------------------------------
E. coli RM9387    -------------------------------------------------------------------------
S. dysenteriae    -------------------------------------------------------------------------

S.Typhimurium     CGTCGACATGCTTAACATCGACCTTCTGATTAAGATCCGCGATGGTAACGTCAAAGACATTCGGCAGGTTGGTCCAGAC
S. Newport        CGTCGACATGCTTAACATCGACCTTCTGATTAAGATCCGCGATGGTAACGTCAAAGACATTCGGCAGGTTGGTCCAGAC
E. coli ED1a      -------------------------------------------------------------------------------
E. coli RM9387    -------------------------------------------------------------------------------
S. dysenteriae    -------------------------------------------------------------------------------

S.Typhimurium     ATGTTCGTTGGAAAAGTAGAGCAGATATTGAGCGGATTACGCGATAGCGGCTGGATAGTCATTAAAAGGGATTTGCTTG
S. Newport        ATGTTCGTTGGAAAAGTAGAGCAGATATTGAGCGGATTACGCGATAGCGGCTGGATAGTCATTAAAAGGGATTTGCTTG
E. coli ED1a      -------------------------------------------------------------------------------
E. coli RM9387    -------------------------------------------------------------------------------
S. dysenteriae    -------------------------------------------------------------------------------

S.Typhimurium     CTGAGAAGCTGGCGACGTGGTGA
S. Newport        CTGAGAAGCTGGCGACGTGGTGA
E. coli ED1a      -----------------------
E. coli RM9387    -----------------------
S. dysenteriae    -----------------------
```
