# Supplementary material for: Gifsy-1 Prophage IsrK with Dual Function as Small and Messenger RNA Modulates Vital Bacterial Machineries
Source: PLoS Genet. 2016 Apr 8;12(4):e1005975. doi: 10.1371/journal.pgen.1005975 (PMC4825925; doi:10.1371/journal.pgen.1005975)
Supplement: S8 Fig — The signs represents full identity (*) conservation (.) and semi conservation (:). Red asterisks represent stop codons. In S. dysenteriae orf45 seem to be fused to anrP (287 amino acids). (PDF) [file pgen.1005975.s008.pdf]

ORF45 amino acid conservation

```
S. Typhimurium    MVGRMGEPKGSPVGDPVTPTLYVSPPNPIGVGGGDKQNNHRRASL* 45 AA
S. Newport        MVGRMGEPKGSPVGDPVTPTLYVSPPNPIGVGGGDKQNNHRRASL* 45 AA
E. coli ED1a      MVGRIGELKSSPVGFPVVPNPVRLATR*------------------ 27 AA
E. coli RM9387    MAGCVGEPKGSPVSVPGSSNPAQFATMMIGT*-------------- 31 AA
S. dysenteriae    MVGCVGEPKGSPVSVPGSLNPAQLATRLLRPEGGDNDQMYGVIVM-
Conservation      *.*: ********.
```

AnrP amino acid conservation

```
S. Typhimurium  MTTQISVETLSPITHNQIPVITTELLAHLYGTKIKNISDNFLNNTTRFVVGKHFFKIEKNELREFKNRP--ETIGLVGKNARS
S. Newport      MTTQISVETLSPITHNQIPVITTELLAHLYGTKIKNISDNFLNNTTRFVVGKHFFKIEKNELREFKNRP--ETIGLVGKNARS
E. coli ED1a    MTTQVSVETLSTITYKQIPVITTELLAHLYGTEAIRIRQNHHENKGRFIEEKHFFKLEGETLREFKHRVAFNYSVKIARNVRS
E. coli RM9387  MTTQISVDTLPAITHNQIPVITTELLAQLYGTKIKNISDNFLNNTTRFVVGKHYFKIEKNELREFKNRP--ETIGLVGKNARS
S. dysenteriae  MLTQISVDTLPAITHNQIRVITTELLAQLYGTKIKNISDNFLNNTMRFVVGKHYFKIEKNELREFKNRP--ETIGLVGKNARS
Conservation    * **:**:**..**::**  ********:****: .* :*. :*. **:  **:**:* : *****:*   :    :..*.**

S. Typhimurium  LILWTERGAARHAKM-LETDQAWEVFEKLEDCYFSQTLPSPTRQVQPAVDMLNIDLLIKIRDGNVKDIRQVGPDMFVGKVEQ
S. Newport      LILWTERGAARHAKM-LETDQAWEVFEKLEDCYFSQTLPSPTRQVQPAVDMLNIDLLIKIRDGNVKDIRQVGPDMFVGKVEQ
E. coli ED1a    LILWTERGAARHAKM-LETDRAWEVFEKLEDCYFSQ----------------------------------------------
E. coli RM9387  LILWTERGAARHAKMTLETDQAWEVFEKLEDCYFSHKQPPAT----------------------------------------
S. dysenteriae  LILWTERGAARHAKM-LETDQAWDVFEKLEDCYFSQ----------------------------------------------
Conservation    *************** ****.**:***********:

S. Typhimurium  ILSGLRDSGWIVIKRDLLAEKLATW*  187 AA
S. Newport      ILSGLRDSGWIVIKRDLLAEKLATW*  187 AA
E. coli ED1a    -------------------------   223 AA
E. coli RM9387  -------------------------   187 AA
S. dysenteriae  -------------------------   287 AA
```
