# Supplementary material for: Gifsy-1 Prophage IsrK with Dual Function as Small and Messenger RNA Modulates Vital Bacterial Machineries
Source: PLoS Genet. 2016 Apr 8;12(4):e1005975. doi: 10.1371/journal.pgen.1005975 (PMC4825925; doi:10.1371/journal.pgen.1005975)
Supplement: S1 Table — (DOCX) [file pgen.1005975.s016.docx]

**S1 Table. LacZ assays**

Mutations affecting the stability of structure B and/or interaction with IsrK*

| Genetic elements | PBAD | PBAD-IsrK | Fold increase |
| --- | --- | --- | --- |
| P*isrK*-*isrK-orf45-anrP'-lacZ* | 3±1 | 417±66 | 139 |
| *orf45-*G114A | 9±0 | 578±58 | 64 |
| *orf45-*G173A | 16±0 | 341±137 | 21 |
| *orf45-*C175U | 8±0 | 319±9 | 40 |
| *orf45-*G114A C175U | 6±0 | 265±25 | 44 |

*Average (miller units) of 14 (wild type) and two independent assays (mutants)
