# Supplementary material for: Gifsy-1 Prophage IsrK with Dual Function as Small and Messenger RNA Modulates Vital Bacterial Machineries
Source: PLoS Genet. 2016 Apr 8;12(4):e1005975. doi: 10.1371/journal.pgen.1005975 (PMC4825925; doi:10.1371/journal.pgen.1005975)
Supplement: S2 Table — (DOCX) [file pgen.1005975.s017.docx]

**S2 Table. Strains**

| Strain | Relevant genotype | Source or reference | Lab stock | | |
| --- | --- | --- | --- | --- | --- |
| SL1344 | *S. typhimurium , hisG46* | Lab collection | | A-112 |  |
|  | SL1344 ΔP*isrK::cat* | This study | | A-593 |  |
|  | SL1344 ΔP*isrK::frt* | This study | | A-595 |  |
|  | SL1344 Δ*isrJ::frt* | [6] | | A-534 |  |
|  | SL1344 ΔP*isrK::cat,* Δ*isrJ::frt*) | This study | | A-622 |  |
|  | SL1344 Δ(P*isrK* to *isrJ::frt)* | This study | | A-624 |  |
|  | SL1344 Δ*anrP::kan* | This study | | A-682 |  |
|  | SL1344 Δ*anrP::frt* | This study | | A-686 |  |
|  | SL1344 Δ*antQ::cat* | This study | | A-684 |  |
|  | SL1344 Δ*antQ::frt* | This study | | A-688 |  |
|  | SL1344 Δ(SL2575*-*SL2576)::*cat* | This study | | A-691 |  |
|  | SL1344 Δ(SL2575*-*SL2576)*::frt* | This study | | A-693 |  |
|  | SL1344 Δ(SL2575*-*SL2576)*::kan* | This study | | A-828 |  |
|  | SL1344 Δ*antQ::cm* Δ(SL2575*-*SL2576)*::kan* | This study | | A-829 |  |
|  | SL1344 Δ(*antQ* to SL2576)*::frt* | This study | | A-830 |  |
|  | SL1344 *orf45*-SPA *kan* | This study | | A-821 |  |
|  | SL1344 *anrP*-SPA *kan* | This study | | A-847 |  |
| LT2 | F112/SH6749A Δ(*malB*) | TS736 [62] | | A-306 |  |
| MC4100 | *E. coli F- araD139 Δ(argF-lac)U169 rpsL150 relA1 flbB5301 deoC1 ptsF25 rbsR* | Lab collection | | A-2 |  |
| RW118 | *thr-1 leuB6 proA2 hisG4 argE3 thi-1 lacY1 galK2 araD139 xyl-5 mtl-1 tsx-33 rpsL31 supE44* *sulA211 pyrD+* | [55] | | A-499 |  |
| MG1655 | *E. coli K-12 F- lambda- ilvG- rfb-50 rph-1* | Lab collection | | A-724 |  |
| MDS42 | MG1655 *fhuACDB(del) endA(del) +* deletion of 699 additional genes, including all IS elements and cryptic prophages | [28] | | A-807 |  |
