# Supplementary material for: Gifsy-1 Prophage IsrK with Dual Function as Small and Messenger RNA Modulates Vital Bacterial Machineries
Source: PLoS Genet. 2016 Apr 8;12(4):e1005975. doi: 10.1371/journal.pgen.1005975 (PMC4825925; doi:10.1371/journal.pgen.1005975)
Supplement: S3 Table — (DOCX) [file pgen.1005975.s018.docx]

**S3 Table. Plasmids**

1. General Plasmids

| Plasmids |  | Genetic elementsa | Origin | Marker | Source or reference |
| --- | --- | --- | --- | --- | --- |
| pKK177-3 |  | P*tac* | ColE1 | Amp^R^ | [63] |
| pSA10 |  | P*tac lacI* | ColE1 | Amp^R^ | Lab collection |
| pJO244 |  | PBAD | ColE1 | Amp^R^ | [64] |
| pRI |  | P*tac* | ColE1 | Amp^R^ | [65] |
| pEF21 |  | PBAD | p15A | Cm^R^ | [64] |
| pACYC184 |  |  | p15A | Cm^R^ Tet^R^ | Lab collection |
| pGEM3 |  |  | ColE1 | Amp^R^ | Lab collection |
| pJL148 |  |  |  | Kan^R^ | [27] |
| pZA31 |  | PL*tetO-1* | P15A | Cm^R^ | [57] |
| pZS*24 |  |  | pSC101* | Kan^R^ | [57] |

1. Plasmids constructed in this study

| Plasmids | Constructionb | Genetic elementsa | Origin | Marker |
| --- | --- | --- | --- | --- |
| pSA67 | pBOG551 | *lacZ* (transcription fusion) | pSC101* | Kan^R^ |
| pSA68 | pBOG552 | *'lacZ* (translation fusion) | pSC101* | Kan^R^ |
| pSA69 | pRI (1364-1365) | P*tac-isrK* | ColE1 | Amp^R^ |
| pSA70 | pJO244 (1364-1365) | PBAD*-isrK* | ColE1 | Amp^R^ |
| pSA70 C18U | pJO244 (1364-1365) | PBAD*-isrK*C18U | ColE1 | Amp^R^ |
| pSA70 G28A | pJO244 (1364-1365) | PBAD*-isrK*G28A | ColE1 | Amp^R^ |
| pSA70 G31A | pJO244 (1364-1365) | PBAD*-isrK*G31A | ColE1 | Amp^R^ |
