# Supplementary material for: Gifsy-1 Prophage IsrK with Dual Function as Small and Messenger RNA Modulates Vital Bacterial Machineries
Source: PLoS Genet. 2016 Apr 8;12(4):e1005975. doi: 10.1371/journal.pgen.1005975 (PMC4825925; doi:10.1371/journal.pgen.1005975)
Supplement: S4 Table — (DOCX) [file pgen.1005975.s019.docx]

**S4 Table. Primers**

Oligonucleotides used for cloning

| Primer | Primer sequence (5'-3') | Usea |
| --- | --- | --- |
| 1364 | CCGAATTCACGCCAGCAAAATCTG | *isrK* (EcoRI +) |
| 1365 | AGCCAAGCTTCCCACCATAATTCAAATGCGCG | *isrK* (HindIII -) |
| 1544 | GCGAATTCATGAACACTCAATACCTCCAG | *antQ* (EcoRI +) |
| 1510 | CGGTCGACCCGGATAACTGTTTGTGTC | *antQ* (SalI -) |
| 1512 | GCGAATTCAACCTTCACCAGACACT | *isrK-orf45-anrP* (EcoRI +) |
| 1703 | CGGGATCCTCAGCAAGCAAATCCCT | *isrK-orf45-anrP* (BamHI -) |
| 2032 | 3’- AACTGCAGAGTAGGACAAATCCGCC-5’ | pZS*24 origin (PstI +) |
| 2042 | 3**'**-ACGCGTCGACGGACTCTGGGGTTCGA-5**'** | pZS*24 origin (SalI -) |
| 1872 | GAAGATCTGGCGTCTGTCATTCGAC | *rho* (BglI +) |
| 1862 | CCCAAGCTTGTGTTACTCTGTAGCGC | *rho* (HindIII -) |
| 1884 | AACTGCAGAGCCAGACTGAGACATG | *srmB* (PstI +) |
| 1885 | CCCAAGCTTATGGAGGCGTGTTTTAC | *srmB* (HindIII -) |
| 1893 | GCGAATTCATGACCACTCAAATCTCTG | *anrP* (EcoRI +) |
| 1897 | CCCTGCAGAATATAGCCGTTTTAATCCAG | *anrP* (PstI -) |
| 1907 | GCTCTAGAGTGACCTTCATAGTGCG | *rnhA* (XbaI +) |
| 1908 | CGGGATCCCTTACGCTTAATCCGCTG | *rnhA* (BamHI -) |
| 1987 | GCGGTACCATGGGGGAGCCGAAAG | *orf45* (KpnI +) |
| 1988 | TCATAATGACGCCCTCCG | *orf45* (-) |
| 1989 | GCGGTACCTTTCTCCTCTTTAATG | WP-PCR (KpnI +) |
| 1990 | CTCTAGAGGCATCAAATAAAACG | WP-PCR(-) |

aPlus (+) and minus (-) strands are indicated. WP-PCR indicates whole plasmid PCR

Oligonucleotides used for Real-Time PCR

| Primer | Primer sequence (5'-3') | Usea |
| --- | --- | --- |
| 1274 | CCAGCAGCCGCGGTAAT | *rrsA* (+) ^7^ |
| 1275 | TTTACGCCCAGTAATTCCGATT | *rrsA* (-) ^7^ |
| 1744 | CTCAATACCTCCAGTATGTACG | *antQ* (+) |
| 1745 | GGGGATAGTTTTTCGTGTC | *antQ* (-) |
| 1869 | AGGCCATTACAGGAGTACG | SL2581 (+) |
| 1870 | CTGATTTGTAGACGTTGCAG | SL2581 (-) |

aPlus (+) and minus (-) strands are indicated

Oligonucleotides used for construction of strains

| Primer | Primer sequence (5'-3') | Usea |
| --- | --- | --- |
| 1377 | TCGCGTTCGGAACAGGTGGCTTTCAACCTATACGCAATGGGAATTAGCCATGGTCC | P*isrK* (*cat* +) |
| 1378 | CTCTGTCGCGGTGAAATTCCGGGACGCCAATCCCGATGTAGGCTGGAGCTGCTTC | P*isrK* (*cat* -) |
| 860 | CGTCTTGATCAGGCGTC | ΔP*isrK* |
| 1197 | TCGCGGCGTGTCTCTGTC | ΔP*isrK* |
| 1132 | CCGAATTCGACGCACTTCTGAAAGGC | Δ(P*isrK* to *isrJ*) |
| 1571 | TGGTGATAAACAAAACAATCACCGGAGGGCGTCATTATGTGTAGGCTGGAGCTGCTTC | Δ*anrP* (*kan* +) |
| 1572 | TTTAATCCAGTTTTGCAATCACCACGTCGCCAGCTTCTCATGGGAATTAGCCATGGTCC | Δ*anrP* (*kan* -) |
| 1528 | CCCTGCAGTCGGTGATCTGGTTACG | Δ*anrP* |
| 1529 | AGCCAAGCTTAATATAGCCGTTTTAATCCAG | Δ*anrP* |
| 1573 | CAATTTTGTTCGTATTTATCAGTTCACAGGATTTACTCAATGGGAATTAGCCATGGTCC | Δ*antQ* (*cat* +) |
| 1458 | CGCATGGTCAGCCAGTGTCTGGTGAAGGTTTCTGACTGTAGGCTGGAGCTGCTTC | Δ*antQ* (*cat* -) |
| 1542 | CCCTGCAGGTTGACTACCGCATCCT | Δ*antQ* |
| 1486 | AGCCAAGCTTCCGGATAACTGTTTGTGTC | Δ*antQ* |
| 1615 | GCGAAATCGCAATTAGCGCACTCAAAAAACGTGCAGGGGTTAATGGGAATTAGCCATGGTCC | ΔSL2575-2576 (*cat* +) |
| 1616 | AAATACTTCAGTTTTTCCTGATCGCTGATGATTCCGGCGCGGTGTAGGCTGGAGCTGCTTC | ΔSL2575-2576 (*cat* -) |
| 2149 | GCGAAATCGCAATTAGCGCACTCAAAAAACGTGCAGGGGTTATGTAGGCTGGAGCTGCTTC | ΔSL2575-2576 (*kan* +) |
| 2150 | AAATACTTCAGTTTTTCCTGATCGCTGATGATTCCGGCGCGGATGGGAATTAGCCATGGTCC | ΔSL2575-2576 (*kan* -) |
| 1617 | TCGGTGTGGACAAGCTG | Δ(SL2575-2576) |
| 1618 | GCGTCCGGATGTACTCC | Δ(SL2575-2576) |
| 1761 | CCGCATATACCGCCGTC | Δ(*antQ* to SL2575-2576) |
| 2122 | GCGGTGGTGATAAACAAAACAATCACCGGAGGGCGTCATTATCCATGGAAAAGAGAAG | *orf45-*SPA (+) |
| 2123 | GTTATGGGTAATCGGGGAAAGAGTTTCGACAGAGATTTGAGTGGTCAGTTCCTATTCCGAAG  TTC | *orf45-*SPA (-) |
| 1987 | GCGGTACCATGGGGGAGCCGAAAG | *orf45-*SPA insertion |
| 2227 | GGATGAGATTTTCTTAAAGCGG | and sequence |
| 2209 | GGCTGGATAGTCATTAAAAGGGATTTGCTTGCTGAGAAGCTGGCGACGTGGTCCATGGAAAA  GAGAAG | *anrP-*SPA (+) |
| 2210 | CAGATTTACATAAAATATAGCCGTTTTAATCCAGTTTTGCAATCAGTTCCTATTCCGAAGTTC | *anrP-*SPA (-) |
| 839 | TCGGCAGGTTGGTCTAG | *anrP-*SPA insertion |

aPlus (+) and minus (-) strands are indicated

Oligonucleotides used for site-directed mutagenesis

| Primer | Primer sequence (5'-3') | Usea,b,c |
| --- | --- | --- |
| 2107 | TGGTGTCGGGATTGGCGTC | G18U (+) |
| 2108 | GATTTTGCTGGCGTACTG | G18U (-) |
| 2129 | CCAATCCCGACGCCAG | G31U (+) |
| 2240 | CATCCCGGAATTTCACCG | G31U (-) |
| 2109 | CTGGTGGGGCGTATGGG | A107C (+) |
| 2110 | AATTCAAATGCGCGTATAC | A107C (-) |
| 1942 | AAAGGGGAGCCGAAAGG | UG120-121AA (+) |
| 2063 | ACGCCCCACTATAATTC | UG120-121AA (-) |
| 2085 | TAGGGGGAGCCGAAAG | AU119-120UA (+) |
| 2086 | ACGCCCCACCATAATTC | AU119-120UA (-) |
| 2111 | TATAGGGGAGCCGAAAG | G121A (+) |
| 2112 | CGCCCCACCATAATTC | G121A (-) |
| 2225 | TAACCCTGTACGTCTCACC | C162U (+) |
| 2226 | GCGTAACCGGATCACC | C162U (-) |
| 2247 | GGCATAACCGGATCACC | C159U (+) |
| 2242 | AACCCTGTACGTCTCAC | C159U (-) |
| 2113 | AGCGTATGGGGGAGCCG | G114A (+) |
| 2114 | CCACCATAATTCAAATGCG | G114A (-) |
| 2105 | ATCTCACCACCCAATCC | G173A (+) |
| 2106 | GTACAGGGTTGGCGTAAC | G173A (-) |
| 2234 | TTCACCACCCAATCCG | C175U (+) |
| 2235 | ACGTACAGGGTTGGCG | C175U (-) |

aPlus (+) and minus (-) strands are indicated

bAll mutant constructs are based on 785 nt fragment (1512-1703) consisting P*isrK-isrK-orf45-anrP'-'lacZ*

cPositions of the mutations or deletions are relative to the transcription start site of IsrK transcript

Oligonucleotides used for *in vitro* RNA synthesis

| Primer | Primer sequence (5'-3') | fragmenta,b |
| --- | --- | --- |
| 1904 | CGAAATTAATACGACTCACTATAGACGCCAGCAAAATCTGG | PT7- *isrK-orf45*1-217 (+)  PT7-*isrK-orf45-anrP*1-785 (+)  PT7- *isrK*1-90 (+) |
| 1948  1703 | CGGGATCCTTGTTTATCACCACCGC  CGGGATCCTCAGCAAGCAAATCCCT | PT7- *isrK-orf45*1-217 (-)  PT7-*isrK-orf45-anrP*1-785 (-) |
| 2256 | TACAAACGACAATAAAAAACACG | PT7- *isrK*1-90 (-) |
| 2316  2317 | CGAAATTAATACGACTCACTATAGGGACAGGGGGTGGTGAGACGTACAGGG  TATGGTGGGGCGTATGGGG | PT7- *anti*-STnc1160 59-1 (+)  PT7- *anti*-STnc1160 59-1 (-) |

aPlus (+) and minus (-) strands are indicated

bThe position of the last nucleotide of the fragment relative to the transcription start site of IsrK is indicated. 1-217 indicates the length of RNA obtained

Oligonucleotides used for northern and native analysis

| Primer | Primer sequence (5'-3') | Use |
| --- | --- | --- |
| 1197 | TCGCGGCGTGTCTCTGTC | IsrK |
| 1471 | GGTCGACGATTGAAGC | IsrJ |
| 459 | GAGACCCCACACTACCATC | 5S RNA |
| 1948 | CGGGATCCTTGTTTATCACCACCGC | ORF45 |
