# Supplementary material for: Gifsy-1 Prophage IsrK with Dual Function as Small and Messenger RNA Modulates Vital Bacterial Machineries
Source: PLoS Genet. 2016 Apr 8;12(4):e1005975. doi: 10.1371/journal.pgen.1005975 (PMC4825925; doi:10.1371/journal.pgen.1005975)
Supplement: S1 Text — (DOCX) [file pgen.1005975.s020.docx]

**Additional References**

Additional references only cited in the supporting information (S2 and S3 Tables)

**62.** Palva ET, Liljeström P, Harayama S (1981) Cosmid cloning and transposon mutagenesis in *Salmonella typhimurium* using phage lambda vehicles. Mol Gen Genet 181(2):153-7. PMID: 6268936.

**63.** Schlosser-Silverman E, Elgrably-Weiss M, Rosenshine I, Kohen R, Altuvia S (2000) Characterization of *Escherichia coli* DNA lesions generated within J774 macrophages. J Bacteriol 182(18):5225-30. PMID: 10960109.

**64.** Guzman LM, Belin D, Carson MJ, Beckwith J (1995) Tight regulation, modulation, and high-level expression by vectors containing the arabinose PBAD promoter. J Bacteriol 177(14):4121-30. PMID: 7608087.

**65.** Opdyke JA, Kang JG, Storz G (2004) GadY, a small-RNA regulator of acid response genes in *Escherichia coli*. J Bacteriol 186(20):6698-705. doi: 10.1128/jb.186.20.6698-6705.2004. PMID: 15466020.
