# Supplementary material for: Correction: Gifsy-1 Prophage IsrK with Dual Function as Small and Messenger RNA Modulates Vital Bacterial Machineries
Source: PLoS Genet. 2017 Apr 14;13(4):e1006725. doi: 10.1371/journal.pgen.1006725 (PMC5391912; doi:10.1371/journal.pgen.1006725)
Supplement: S3 Table — (DOCX) [file pgen.1006725.s001.docx]

**Table S3. Plasmids**

1. General Plasmids

| Plasmids |  | Genetic elementsa | Origin | Marker | Source or reference |
| --- | --- | --- | --- | --- | --- |
| pKK177-3 |  | P*tac* | ColE1 | Amp^R^ | [63] |
| pSA10 |  | P*tac lacI* | ColE1 | Amp^R^ | Lab collection |
| pJO244 |  | PBAD | ColE1 | Amp^R^ | [64] |
| pRI |  | P*tac* | ColE1 | Amp^R^ | [65] |
| pEF21 |  | PBAD | p15A | Cm^R^ | [64] |
| pACYC184 |  |  | p15A | Cm^R^ Tet^R^ | Lab collection |
| pGEM3 |  |  | ColE1 | Amp^R^ | Lab collection |
| pJL148 |  |  |  | Kan^R^ | [27] |
| pZA31 |  | PL*tetO-1* | P15A | Cm^R^ | [57] |
| pZS*24 |  |  | pSC101* | Kan^R^ | [57] |

1. Plasmids constructed in this study

| Plasmids | Constructionb | | Genetic elementsa | Origin | | Marker |
| --- | --- | --- | --- | --- | --- | --- |
| pSA67 | pBOG551 | | *lacZ* (transcription fusion) | pSC101* | | Kan^R^ |
| pSA68 | pBOG552 | | *'lacZ* (translation fusion) | pSC101* | | Kan^R^ |
| pSA69 | pRI (1364-1365) | | P*tac-isrK* | ColE1 | | Amp^R^ |
| pSA70 | pJO244 (1364-1365) | | PBAD*-isrK* | ColE1 | | Amp^R^ |
| pSA70 C18U | pJO244 (1364-1365) | | PBAD*-isrK*C18U | ColE1 | | Amp^R^ |
| pSA70 G28A | pJO244 (1364-1365) | | PBAD*-isrK*G28A | ColE1 | | Amp^R^ |
| pSA70 G31A | pJO244 (1364-1365) | | PBAD*-isrK*G31A | ColE1 | | Amp^R^ |
| pSA71 | pKK177*-*3*-lacI* (1893-1897) | P*tac-anrP-lacI* | | | ColE1 | Amp^R^ |
| pSA72 | pKK177*-*3*-lacI* (1544-1510) | P*tac-antQ-lacI* | | | ColE1 | Amp^R^ |
| pSA74 | pACYC184 (1907-1908) | P*rnhA-rnhA* | | | p15A | Cm^R^ |
| pSA75 | pEF21 (1884-1885) | PBAD*-srmB* | | | p15A | Cm^R^ |
| pSA76 | pACYC184 (1872-1862) | P*rho-rho* | | | p15A | Cm^R^ |
| pSA77 | pGEM3 (1512-1703) | P*isrK-isrK-orf43-anrP* | | | ColE1 | Amp^R^ |
| pSA77 A107C | pGEM3 (1512-1703) | P*isrK-isrK-orf43*A107C*-anrP* | | | ColE1 | Amp^R^ |
| pSA77 UG120-121AA | pGEM3 (1512-1703) | P*isrK-isrK-orf43*UG120-121AA*-anrP* | | | ColE1 | Amp^R^ |
| pSA77 AU119-120UA | pGEM3 (1512-1703) | P*isrK-isrK-orf43*AU119-120UA*-anrP* | | | ColE1 | Amp^R^ |
| pSA77 A107C G121A | pGEM3 (1512-1703) | P*isrK-isrK -orf43*A107C G121A*-anrP* | | | ColE1 | Amp^R^ |
| pSA77 G28A | pGEM3 (1512-1703) | P*isrK-isrK*G28A*-orf43-anrP* | | | ColE1 | Amp^R^ |
| pSA77 C162U | pGEM3 (1512-1703) | P*isrK-isrK-orf43*C162U*-anrP* | | | ColE1 | Amp^R^ |
| pSA77 G28A C162U | pGEM3 (1512-1703) | P*isrK-isrK -orf43*G28A C162U*-anrP* | | | ColE1 | Amp^R^ |
| pSA77 G31A | pGEM3 (1512-1703) | P*isrK-isrK*G31A*-orf43-anrP* | | | ColE1 | Amp^R^ |
| pSA77 C159U | pGEM3 (1512-1703) | P*isrK-isrK-orf43* C159U *-anrP* | | | ColE1 | Amp^R^ |
| pSA77 G31A C159U | pGEM3 (1512-1703) | P*isrK-isrK -orf43*G31A C159U*-anrP* | | | ColE1 | Amp^R^ |
| pSA77 G114A | pGEM3 (1512-1703) | P*isrK-isrK-orf43*G114A*-anrP* | | | ColE1 | Amp^R^ |
| pSA77 G173A | pGEM3 (1512-1703) | P*isrK-isrK-orf43*G173A*-anrP* | | | ColE1 | Amp^R^ |
| pSA77 C175U | pGEM3 (1512-1703) | P*isrK-isrK-orf43*C175U*-anrP* | | | ColE1 | Amp^R^ |
| pSA77 G114A C175U | pGEM3 (1512-1703) | P*isrK-isrK -orf43*G114A C175U*-anrP* | | | ColE1 | Amp^R^ |
| pSA78 | pGEM3 (1364-1703) | *isrK-orf43-anrP* | | | ColE1 | Amp^R^ |
| pSA79 | pGEM3 (1512-2010) | P*isrK-isrK-orf43* | | | ColE1 | Amp^R^ |
| pSA80 | pBOG551 (1512-1703) | P*isrK-isrK-orf43-anrP'-lacZ* | | | pSC101 | Kan^R^ |
| pSA81 | pBOG552 (1512-1703) | P*isrK-isrK-orf43-anrP'-'lacZ* | | | pSC101 | Kan^R^ |
| pSA81 A107C | pBOG552 (1512-1703) | P*isrK-isrK-orf43*A107C*- anrP'-'lacZ* | | | pSC101 | Kan^R^ |
| pSA81 UG120-121AA | pBOG552 (1512-1703) | P*isrK-isrK-orf43*UG120-121AA*- anrP'-'lacZ* | | | pSC101 | Kan^R^ |
| pSA81 AU119-120UA | pBOG552 (1512-1703) | P*isrK-isrK-orf43*AU119-120UA*- anrP'-'lacZ* | | | pSC101 | Kan^R^ |
| pSA81 A107C G121A | pBOG552 (1512-1703) | P*isrK-isrK -orf43*A107C G121A*- anrP'-'lacZ* | | | pSC101 | Kan^R^ |
| pSA81 G28A | pBOG552 (1512-1703) | P*isrK-isrK*G28A*-orf43-anrP'-'lacZ* | | | pSC101 | Kan^R^ |
| pSA81 C162U | pBOG552 (1512-1703) | P*isrK-isrK-orf43*C162U*-anrP'-'lacZ* | | | pSC101 | Kan^R^ |
| pSA81 G28A C162U | pBOG552 (1512-1703) | P*isrK-isrK -orf43*G28A C162U*-anrP'-'lacZ* | | | pSC101 | Kan^R^ |
| pSA81 G31A | pBOG552 (1512-1703) | P*isrK-isrK*G31A*-orf43-anrP'-'lacZ* | | | pSC101 | Kan^R^ |
| pSA81 C159U | pBOG552 (1512-1703) | P*isrK-isrK-orf43*C159U*-anrP'-'lacZ* | | | pSC101 | Kan^R^ |
| pSA81 G31A C159U | pBOG552 (1512-1703) | P*isrK-isrK -orf43*G31A C159U*-anrP'-'lacZ* | | | pSC101 | Kan^R^ |
| pSA81 G114A | pBOG552 (1512-1703) | P*isrK-isrK-orf43*G114A*- anrP'-'lacZ* | | | pSC101 | Kan^R^ |
| pSA81 G173A | pBOG552 (1512-1703) | P*isrK-isrK-orf43*G173A*- anrP'-'lacZ* | | | pSC101 | Kan^R^ |
| pSA81 C175U | pBOG552 (1512-1703) | P*isrK-isrK-orf43*C175U*- anrP'-'lacZ* | | | pSC101 | Kan^R^ |
| pSA81 G114A C175U | pBOG552 (1512-1703) | P*isrK-isrK -orf43*G114A C175U*- anrP'-'lacZ* | | | pSC101 | Kan^R^ |
| pSA82 | pBOG551 (1364-1703) | *isrK-orf43-anrP'-lacZ* | | | pSC101 | Kan^R^ |
| pSA83 | pBOG552 (1364-1703) | *isrK-orf43-anrP'-'lacZ* | | | pSC101 | Kan^R^ |

aPosition of the mutant bases is relative to the transcription start site

bThe numbers in parentheses represent primers used to construct the plasmids
